# Mural cell-derived laminin-α5 plays a detrimental role in ischemic stroke

**DOI:** 10.1186/s40478-019-0676-8

**Published:** 2019-02-18

**Authors:** Abhijit Nirwane, Jessica Johnson, Benjamin Nguyen, Jeffrey H. Miner, Yao Yao

**Affiliations:** 1 Department of Pharmaceutical and Biomedical Sciences, University of Georgia, 240 W Green Street, Athens, GA 30602 USA; 2 Division of Nephrology, Department of Medicine, Washington University School of Medicine, St. Louis, MO USA

**Keywords:** Blood-brain barrier, Ischemic stroke, MCAO, Mural cells, Laminin

## Abstract

At the blood-brain barrier (BBB), laminin-α5 is predominantly synthesized by endothelial cells and mural cells. Endothelial laminin-α5 is dispensable for BBB maintenance under homeostatic conditions but inhibits inflammatory cell extravasation in pathological conditions. Whether mural cell-derived laminin-α5 is involved in vascular integrity regulation, however, remains unknown. To answer this question, we generated transgenic mice with laminin-α5 deficiency in mural cells (α5-PKO). Under homeostatic conditions, no defects in BBB integrity and cerebral blood flow (CBF) were observed in α5-PKO mice, suggesting that mural cell-derived laminin-α5 is dispensable for BBB maintenance and CBF regulation under homeostatic conditions. After ischemia-reperfusion (MCAO) injury, however, α5-PKO mice displayed less severe neuronal injury, including reduced infarct volume, decreased neuronal death, and improved neurological function. In addition, α5-PKO mice also showed attenuated vascular damage (milder BBB disruption, reduced inflammatory cell infiltration, decreased brain edema, and diminished hemorrhagic transformation). Mechanistic studies revealed less severe tight junction protein (TJP) loss and pericyte coverage reduction in α5-PKO mice after ischemia-reperfusion injury, indicating that the attenuated ischemic injury in α5-PKO mice is possibly due to less severe vascular damage. These findings suggest that mural cell-derived laminin-α5 plays a detrimental role in ischemic stroke and that inhibiting its signaling may have a neuroprotective effect.

**Electronic supplementary material:**

The online version of this article (10.1186/s40478-019-0676-8) contains supplementary material, which is available to authorized users.

## Introduction

The blood-brain barrier (BBB) is a dynamic structure mainly composed of brain microvascular endothelial cells (BMECs), pericytes, astrocytes, and a non-cellular component---the basement membrane (BM) [7, 57, 77]. By tightly regulating substance exchange between the CNS and circulation system, the BBB functions to maintain CNS homeostasis. Accumulating evidence suggests that BBB disruption contributes to the pathogenesis and progression of various neurological disorders [48, 81, 82]. For example, BBB breakdown affects inflammatory cell infiltration and is associated with the development/progression of ischemia-reperfusion injury [15, 32, 75]. It should be noted that the majority of BBB studies focus on its cellular constituents, and the role of the BM in BBB regulation remains largely unknown.

The BM consists of highly organized extracellular matrix proteins synthesized by astrocytes, BMECs, and mural cells, which include both pericytes and vascular smooth muscle cells (vSMCs) [29, 51, 67, 76]. Laminin, the only protein that is absolutely required for BM formation, is a trimer composed of α, β, and γ subunits [20, 51, 76, 77]. Among all five genetic variants of the α subunits, laminin-α4 and -α5 are highly expressed in blood vessels throughout the body [29, 67, 76]. Unlike laminin-α4, which is ubiquitously distributed in the vasculature, laminin-α5 expression shows a patchy pattern at smaller vessels [73]. The major cell types that synthesize laminin-α5 in the vasculature are BMECs and mural cells [26, 29, 46, 62, 65, 67, 80]. Recent studies demonstrated that knockout of laminin-α5 in endothelial cells failed to affect BBB permeability under homeostatic conditions [25, 63]. In TNFα-induced inflammation, however, these mutants showed significantly enhanced neutrophil extravasation in cremaster muscle [63]. In collagenase-induced intracerebral hemorrhage (ICH) model, these mutants displayed exacerbated inflammatory cell infiltration [25]. In addition, in the experimental autoimmune encephalomyelitis (EAE) model, decreased T cell infiltration into the brain and reduced disease susceptibility & severity were observed in laminin-α4 null mice [73], which exhibited compensatory and ubiquitous expression of laminin-α5 along the vasculature [73]. These findings suggest that endothelial laminin-α5 plays an inhibitory role in inflammatory cell extravasation under pathological conditions, although it is dispensable for BBB maintenance under physiological conditions [25, 63]. Whether mural cell-derived laminin-α5 is involved in BBB regulation under physiological and pathological conditions, however, remains unknown. Given that mural cell-derived laminin-α5 is an important component of the BM at the BBB [51, 76], we hypothesize that mural cell-derived laminin-α5 may also contribute to BBB integrity. In this study, we investigated the functions of mural cell-derived laminin-α5 in BBB regulation under homeostatic conditions and in ischemic stroke.

## Materials and methods

### Mice

The experimental protocols were reviewed and approved by the Institutional Animal Care and Use Committee at the University of Georgia and were in accordance with the National Institute of Health Guide for Care and Use of Laboratory Animals. The Animal Research: Reporting In Vivo Experiments (ARRIVE) guidelines for reporting experiments involving animals were strictly followed. Laminin-α5^flox/flox^ mice were generated as described previously [50]. Pdgfrβ-Cre^+^ mice were a generous gift from Dr. Volkhard Lindner. These two transgenic lines were crossed to generate laminin-α5^flox/flox^: Pdgfrβ-Cre^+^ (α5-PKO) mice. Their wildtype littermates were used as controls. In this study, 194 mice (102 control and 92 α5-PKO) were used. All mice were housed in the animal facility at the University of Georgia with free access to water and food.

### Middle cerebral artery occlusion (MCAO)

Eight-week-old control and α5-PKO mice were subjected to 45 min of focal cerebral ischemia produced by transient intraluminal occlusion of the middle cerebral artery using a filament as described previously [49, 68]. Briefly, mice were anesthetized with 2,2,2-tribromoethyl alcohol (250 mg/kg, *i.p*.). A midline neck incision was made and the common carotid artery (CCA), external carotid artery (ECA), and internal carotid artery (ICA) on the right side were carefully isolated. The ECA and CCA were ligated distal to the carotid bifurcation. The ICA was clipped temporarily. A 6–0 silicone monofilament suture (Doccol) with a 0.21 mm diameter was introduced into the CCA via an incision, advanced 9 mm distal to the carotid bifurcation and secured in place. Successful occlusion of the middle cerebral artery was confirmed with the PeriCam PSI HR system (Perimed) based on laser speckle contrast analysis technology. Animals showing diminished blood flow of at least 80% during occlusion with at least 75% recovery of blood flow after reperfusion were used for experimentation. The body temperature was maintained at 37.0 ± 0.5 °C during the surgery using a heating pad. Animals had free access to food and water throughout the reperfusion period. This ischemic model led to ~ 30% and ~ 20% mortality rates for control and α5-PKO mice, respectively.

### Body weight loss and neurological function

The body weight loss was evaluated daily from days 1 to 7 after surgery. Neurological function was assessed using the modified neurological severity scores (mNSS) system, which evaluates motor, sensory, reflex and balance functions, as described previously [16, 38, 58]. Briefly, mice were scored based on their performances in a variety of tests as described in Additional file 1: Table S1. The sum of these scores (0–14) was used to reflect their neurological function after MCAO. Higher scores indicate worse neurological function. Animals were habituated to the testing environment prior to experiments and the investigator who scored the animals was blinded to the genotypes.

### Brain sectioning

Serial sectioning was used in this study. Briefly, 20 μm-thick serial sections were cut with Cryostat (Micro HM 550, Thermo Scientific). Eight sections evenly distributed along the rostral-to-caudal axis were collected from each brain.

### Infarct volume and neuronal death

Brain infarct volume was quantified as infarct volume percentage (%) as described previously [43, 53, 56]. Briefly, cresyl violet-stained brain sections were imaged using the Nikon Eclipse Ti microscope. The areas of the contralateral hemisphere (*C*_*i*_), ipsilateral hemisphere (*I*_*i*_), and ipsilateral non-ischemic region (*N*_*i*_) were determined using the Image J software (NIH), and the infarct volume (%) was calculated as:$\mathrm{Infarctvolume}\left(\%\right)=\left(\frac{\sum \limits_i\left(\left(\frac{I_i-{N}_i}{I_i}\right){C}_i\right)}{2{\sum}_i{C}_i}\right)x100$.

Neuronal death was assessed using Fluoro-Jade C (FJC) staining as described previously [59, 79]. Specifically, the number of FJC^+^ cells was counted in each field. At least 3 random fields from each section, 8 serial sections per brain, and 4 animals were used for quantification.

### BBB permeability

Evans blue (EB) and/or FITC-Dextran (4kD) were used to assess BBB permeability as described previously [15]. Briefly, control and α5-PKO mice were injected retro-orbitally with 80 μl EB (2%, Sigma E2129) and/or 50 μl FITC-Dextran (25 mg/ml, Sigma FD4). For non-ischemic study, FITC-Dextran was allowed to circulate for 12 h. After transcardial perfusion, the brains were collected, homogenized in formamide, and centrifuged at 20,000 rpm for 20 min. The fluorescence intensity of the supernatant was measured using a SpectraMax M2 plate reader (Molecular Devices) at 450/550 nm. Mice without FITC-Dextran injection were used to determine baseline reading, which was subtracted from raw reading to obtain FITC-Dextran leakage. Leakage in α5-PKO mice was normalized to that in controls. For ischemic study, both tracers were injected 4 h before mice were transcardially perfused at each time point after injury. Each brain hemisphere was homogenized in formamide and centrifuged at 20,000 rpm for 20 min. The absorbance and fluorescence intensity of the supernatant were measured using a SpectraMax M2 plate reader at 620 nm and 450/550 nm, respectively. EB or FITC-Dextran leakage was defined as the difference of absorbance or fluorescence intensity between contralateral and ipsilateral hemispheres. Leakage in α5-PKO mice was normalized to that in controls.

### Brain edema

Brain edema was assessed using both brain water content [79] and brain swelling [33] as described previously. Briefly, control and α5-PKO mice were transcardially perfused with PBS. Brains were collected and cut into left and right hemispheres. The weights of each hemisphere before and after drying at 85 °C for 4 h were measured and defined as wet and dry weights, respectively. Brain water content (%) was calculated as (Wet Weight - Dry Weight) / Wet Weight × 100. Brain swelling (%) was calculated as (Final wet weight_ipsi_ – Initial wet weight_ipsi_) / Initial wet weight_ipsi_ × 100. In this equation, final wet weight_ipsi_ is the wet weight of ipsilateral hemisphere. Initial wet weight_ipsi_ is defined as (Wet weight_contra_ / Dry weight_contra_) x Dry weight_ipsi._

### Immunofluorescence analyses

Immunofluorescence analyses were performed according to standard protocols. Briefly, brain sections and/or cells were fixed in 4% PFA for 15 min at room temperature and washed in PBS 3 times. Next, the sections and cells were blocked in blocking buffer (5% normal donkey serum in PBS + 1% BSA + 0.3% Triton X-100) for 2 h at room temperature, followed by incubation with anti-Laminin-α2 (1:400, Sigma L0663), anti-Laminin-α5 (1:800, generated as described in [47]), anti-Smooth Muscle Actin-α (SMA)-FITC (1:1000, Sigma F3777), anti-Hemoglobin (1:200, Cloud-Clone PAB409Mu01), anti-Ly6G (1:200, Biolegend 108,402), anti-CD3 (1:200, eBioscience 14–0032-82), anti-CD68 (1:200, Biolegend 137,002), anti-PDGFRβ (1:200, Cell Signaling 3169S), anti-ZO-1(1:400, ThermoFisher 61–7300), anti-Claudin-5 (1:200, Invitrogen 35–2500), anti-AQP4 (1:200, Millipore AB3594), and anti-CD31 (1:200, BD Bioscience 553,370) antibodies overnight at 4 °C. After extensive washes in PBS, the sections and/or cells were incubated with the following secondary antibodies: Alexa Fluor-488 conjugated donkey anti-rabbit (1:1000, Invitrogen A21206), Alexa Fluor-594 conjugated donkey anti-rabbit (1:1000, Invitrogen A21207), FITC conjugated goat anti-mouse (1:500, BD Pharmingen 554,001), Alexa Fluor-594 conjugated donkey anti-mouse (1:1000, Invitrogen A21203), FITC conjugated goat anti-rat (1:500, BD Pharmingen 554,016), Alexa Fluor-594 conjugated donkey anti-rat (1:1000, Invitrogen A21209), and Alexa Fluor-647 conjugated goat anti-rat (1:1000, Invitrogen A21247) for 2 h at room temperature. Then, the sections and/or cells were washed in PBS 3 times and mounted in Fluoromount-G with DAPI. Images were taken under a Nikon Eclipse Ti microscope or LSM710 confocal microscope. Image processing was performed using ImageJ and Adobe Photoshop.

### Image analyses

Brain angioarchitecture analyses were performed using the open source “Angiotool” software (National Cancer Institute, USA) as described previously [83]. Specifically, CD31-stained brain sections were used for analyses. Vessel length, defined as the sum of Euclidean distances between the pixels of all vessels; vessel density, defined as the percentage of area occupied by vessels inside the explant area; and branching index, defined as the number of vessel junctions per unit area, were computed in the cortex and striatum. Thresholding was applied to remove small particles so that only actual vessels were quantified [24]. For quantification, 8 serial sections along the rostral-to-caudal axis were analyzed for each brain and 4 mice were used. Data in α5-PKO mice were normalized to that in controls.

For pericyte coverage, PDGFRβ- and CD31-positive fluorescent areas were determined using ImageJ area measurement tool. Pericyte coverage was determined as the percentage (%) of PDGFRβ-positive fluorescent area covering CD31-positive capillary area, as previously described [8]. For TJP and AQP4 coverage, ZO-1/Claudin-5/AQP4- and CD31-positive fluorescent areas were determined using ImageJ area measurement tool. ZO-1/Claudin-5/AQP4 coverage was determined as the percentage (%) of ZO-1/Claudin-5/AQP4-positive fluorescent area covering CD31-positive capillary area. For inflammatory cell infiltration, total numbers of Ly6G^+^/CD3^+^/CD68^+^ cells were counted. For hemoglobin staining, mean fluorescence intensity was used. For quantification, at least three random fields from each section, 8 serial sections along the rostral-to-caudal axis for each brain, and 4–5 animals were used. All data analyses were performed on z-projection (10-12 μm) images by a blinded investigator.

For laminin-α5 immunocytochemistry, the percentage of laminin-α5^+^ cells were calculated. For quantification, 6 independent experiments were performed and at least 50 cells were examined in each experiment.

### Brain mural cell isolation

Primary mural cells were isolated from mouse brains using a well-established protocol. Briefly, brains were collected under aseptic conditions. After removing meninges, the brains were minced with a blade and triturated. Brain tissue was then incubated with 0.1% collagenase at 37 °C for 1 h followed by centrifuge at 700 g for 8 min. The pellet was resuspended in 17% sterile dextran solution and centrifuged at 6000 g for 20 min at 4 °C. Blood vessel-containing pellet was washed in DMEM for 3 times and further digested in 1 mg/ml collagenase/dispase (Roche, 11,097,113,001) for 2 h with constant shaking at 37 °C. Next, red blood cells (RBCs) were removed by washing the pellet in RBC lysis buffer. The pellet was resuspended in sorting buffer and passed through a 40 μm cell strainer. The single-cell solution was then stained with anti-CD31-APC (1:100, Biolegend 102,509), anti-CD45-FITC (1:100, Biolegend 103,108), and anti-Pdgfrβ-PE (1:100, eBioscience 12–1402) for 30 min at 4 °C. After extensive wash, the cells were subjected to FACS. Sorted mural cells (Pdgfrβ^+^CD31^−^CD45^−^) were grown in Pericyte Medium (ScienCell, 1201) and used for immunocytochemistry.

### Transmission electron microscopy (TEM)

Eight-week-old control and α5-PKO mice were anesthetized and perfused with PBS followed by 0.1 M sodium cacodylate buffer containing 2% paraformaldehyde and 2% glutaraldehyde. After perfusion, brain tissue was dissected out, fixed overnight, and post-fixed in 1% osmium tetroxide and 1% K-ferrocyanide. Next, the tissue was en bloc stained with 2% uranyl acetate and embedded in resin. Ultra-thin sections were cut on an RMC MT-X microtome (Boeckeler Instruments) and post-stained with 2% uranyl acetate and 1% lead citrate. Sections were examined and photographed using JEOL JEM1011 (JEOL) at 80 kV.

### Western blotting

Cortex and striatum were carefully dissected and immediately homogenized on ice. Total protein concentration was determined using the BCA Protein Assay Kit (Pierce 23,227). Equal amount of protein was loaded and separated in SDS-PAGE and transferred to PVDF membranes (Millipore). Next, the membranes were probed with primary antibodies [anti-Laminin-α2 (1:500, Sigma L0663), anti-Laminin α5 (1:800, generated as described in [47]), Claudin-5 (1:500, ThermoFisher 35–2500), ZO-1 (1:500, ThermoFisher 61–7300), and anti-GAPDH (1:1000, Abcam AB9484)] over night at 4 °C, followed by appropriate horseradish peroxidase-conjugated secondary antibodies [donkey anti-mouse (1:2500, Jackson ImmunoResearch Laboratory 715–035-151), donkey anti-rabbit (1:2500, Jackson ImmunoResearch Laboratory 711–035-152), and donkey anti-rat (1:2500, Jackson ImmunoResearch Laboratory 712–035-153] at room temperature for 1 h. Then, target proteins were visualized using the ChemiDoc Imaging System (Bio-Rad). For quantification, the density of target blots was normalized to that of GAPDH, and the expression of target proteins in α5-PKO brains was normalized to that in control brains. Four animals were used for quantification.

### Statistical analyses

All statistical analyses were performed using the GraphPad Prism 6 software. For normally distributed measurements, unpaired Student’s *t*-test was used to determine statistical significance between two groups, and one-way analysis of variance (ANOVA) followed by Tukey post-hoc test was used for three or more groups. For measurements that are not normally distributed, the non-parametric Mann-Whitney *U* test (two groups) and Kruskal-Wallis test (three or more group) were used. Significance was set at *p* < 0.05. Data were presented as mean ± SD.

## Results

### Laminin-α5 is indeed abrogated in mural cells in α5-PKO mice

**Fig. 1:**
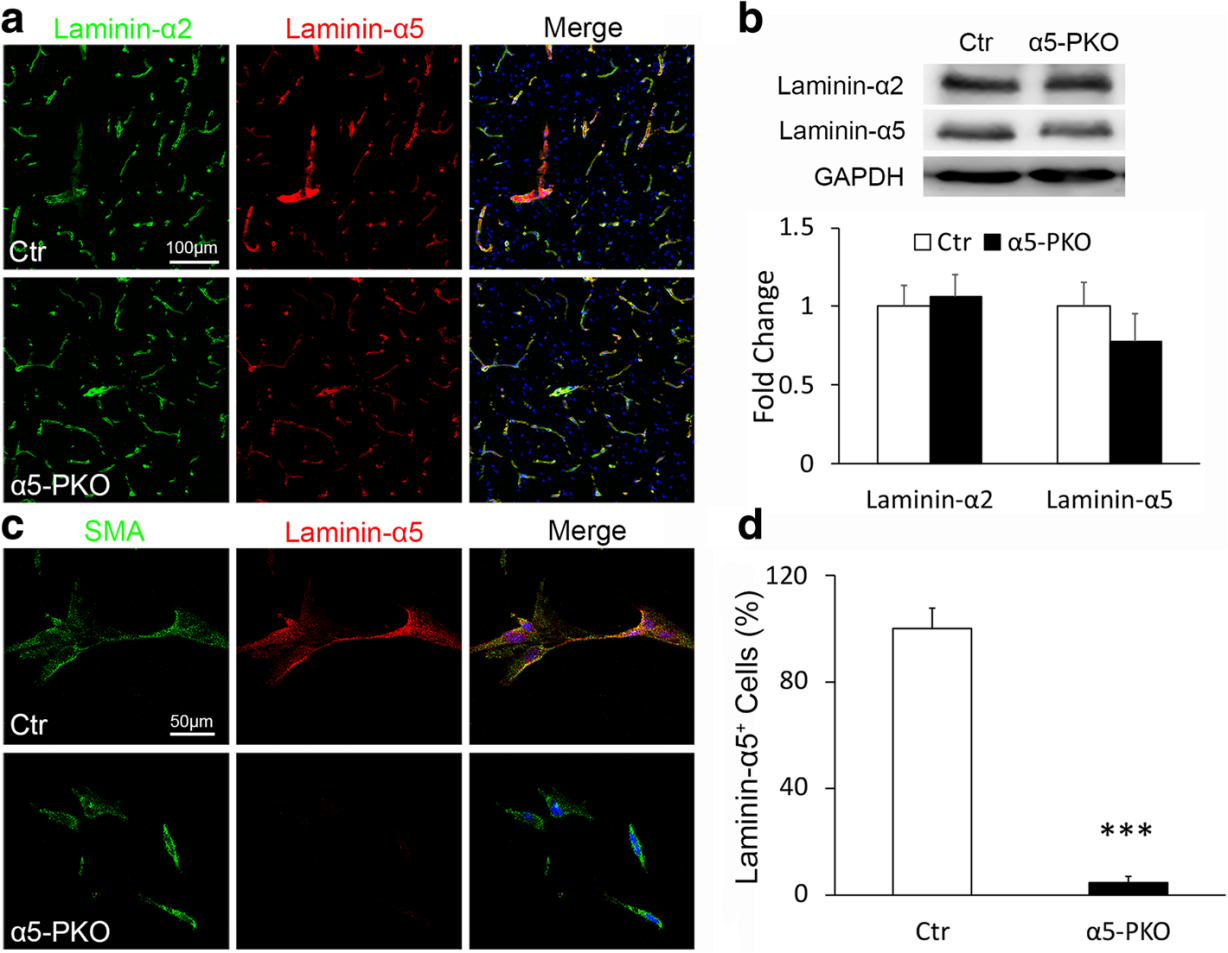
Lama5 expression is abrogated in mural cells in α5-PKO mice. **a** Representative images of laminin-α2 (green) and laminin-α5 (red) staining in the cortex of control and α5-PKO mice. Scale bar = 100 μm. **b** Representative western blotting and quantification of laminin-α2 and laminin-α5 levels in the cortex of control and α5-PKO mice. *n* = 4. **c** Representative images of smooth muscle actin-α (SMA, green) and laminin-α5 (red) staining in primary mural cells isolated from control and α5-PKO brains. Scale bar = 50 μm. **d** Quantification showing the lack of laminin-α5 expression in primary mural cells isolated from α5-PKO brains. *n* = 6 independent experiments with at least 50 cells examined in each experiment. Student’s *t*-test, ***p < 0.001, compared to controls

The α5-PKO mice are born at the expected Mendelian ratio, fail to show gross abnormalities, and have a normal lifespan. Using lineage-tracing technique, we have demonstrated that Pdgfrβ-Cre specifically targets mural cells in the brain [26]. Immunohistochemistry revealed laminin-α2 and laminin-α5 expression in the cortex of both control and α5-PKO mice (Fig. 1a). To quantitatively determine the expression levels of laminin-α2 and laminin-α5, western blot analysis was performed using cortical tissue. As expected, comparable levels of laminin-α2 were found in control and α5-PKO mice (Fig. 1b). Laminin-α5, on the other hand, was slightly reduced in α5-PKO mice, although statistical significance was not reached (Fig. 1b). Similar results were observed in the striatum (not shown). The residual expression of laminin-α5 in α5-PKO brains is probably from endothelial cells, which synthesize laminin-511 and -411 [29, 62, 65]. To further determine if laminin-α5 expression is abrogated in mural cells in α5-PKO mice, we isolated primary mural cells from control and α5-PKO brains using a well-established protocol [9, 26, 78] and examined laminin-α5 expression in these cells. Isolated cells expressed mural cell marker SMA (Fig. 1c), suggesting they were indeed mural cells. Immunocytochemistry revealed laminin-α5 expression in control but not α5-PKO mural cells (Fig. 1c). Quantification showed that almost all control mural cells expressed laminin-α5, whereas more than 95% of α5-PKO mural cells were negative for laminin-α5 (Fig. 1d). These results indicate that laminin-α5 is indeed abrogated in mural cells in α5-PKO mice.

### Brain angioarchitecture is unaffected in α5-PKO mice under homeostatic conditions

To determine if α5-PKO mice have abnormal brain angioarchitecture, we analyzed vessel length, vessel density, and branching index in both cortex and striatum using the “Angiotool” software. None of these parameters showed significant differences in the cortex (Additional file 1: Figure S1) or striatum (not shown) in α5-PKO mice compared to the controls (Additional file 1: Figure S1), strongly suggesting that loss of laminin-α5 in mural cells does not affect brain angioarchitecture.

### BBB integrity and cerebral blood flow (CBF) are unchanged in α5-PKO mice under homeostatic conditions

**Fig. 2:**
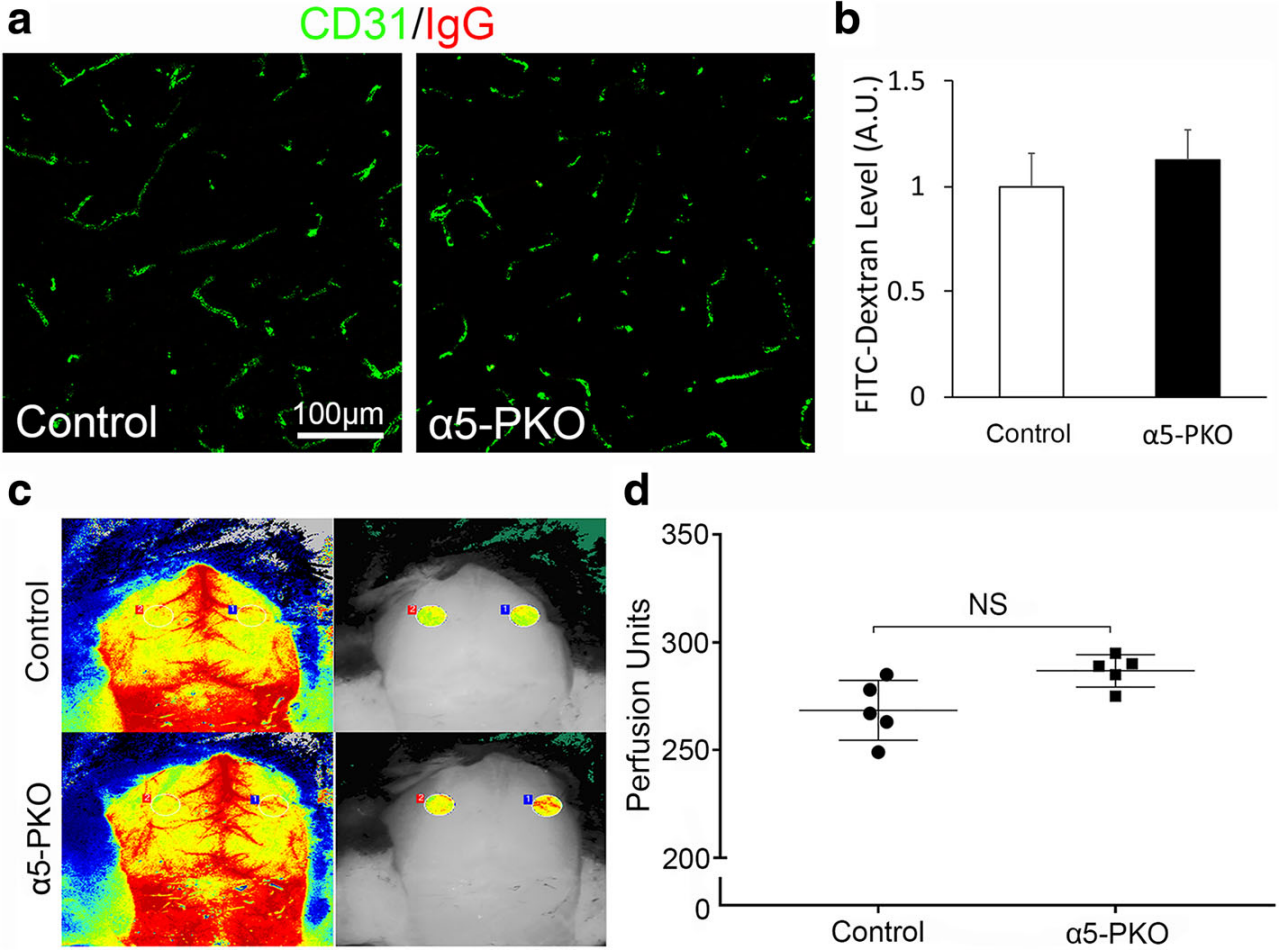
BBB integrity and CBF are unaffected in α5-PKO mice under homeostatic conditions. **a** Representative images of IgG (red) and CD31 (green) staining in the cortex of control and α5-PKO mice. Scale bar = 100 μm. **b** Quantification showing comparable levels of FITC-Dextran in control and α5-PKO brains. *n* = 5. **c** Representative laser speckle images of CBF/brain perfusion in control and α5-PKO brains. **d** Quantification showing similar levels of CBF/brain perfusion in control and α5-PKO brains. *n* = 5

To investigate if BBB integrity is disrupted in α5-PKO mice, IgG leakage was examined by immunohistochemistry. No IgG signal was detected in the cortex (Fig. 2a) or striatum (not shown) of control or α5-PKO mice, suggesting that the BBB is not leaky to molecules with a size of 150kD or above. Next, FITC-Dextran (4kD), a smaller dye, was used to assess BBB integrity. Comparable levels of FITC-Dextran were found in control and α5-PKO brains (Fig. 2b), suggesting intact BBB integrity in α5-PKO mice under homeostatic conditions. To investigate if CBF is altered in α5-PKO mice, real-time CBF in middle cerebral artery regions was measured. Representative laser speckle images of CBF in control and α5-PKO mice are shown in Fig. 2c. Quantification revealed no significant difference in CBF between control and α5-PKO mice (Fig. 2d), suggesting unaffected CBF in α5-PKO mice under homeostatic conditions. Together, these findings suggest that loss of mural cell-derived laminin-α5 does not affect BBB integrity or CBF under homeostatic conditions.

### TJP expression and tight junction structure are unaltered in α5-PKO mice under homeostatic conditions

**Fig. 3:**
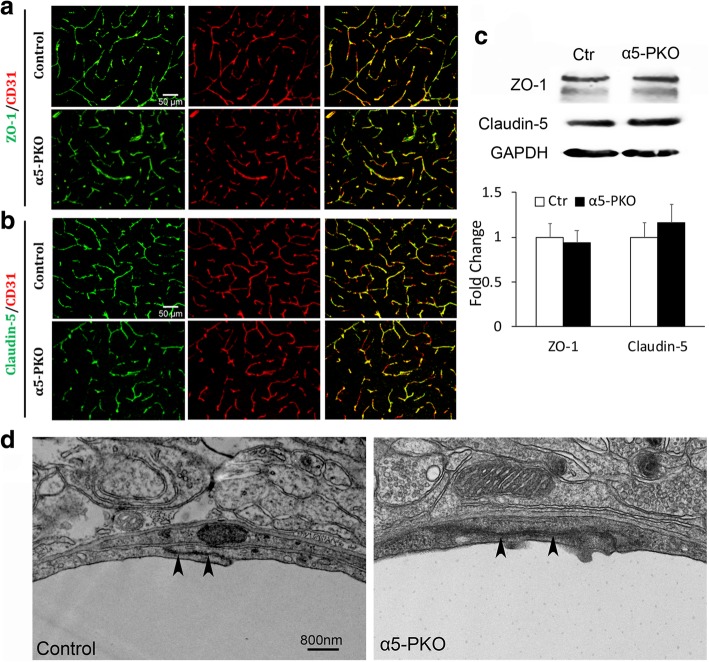
Tight junctions are unaffected in α5-PKO mice under homeostatic conditions. **a,b** Representative images of ZO-1 (green)/CD31 (red) (**a**) and Claudin-5 (green)/CD31 (red) (**b**) staining in the cortex of control and α5-PKO mice. Scale bar = 50 μm. **c** Representative western blotting and quantification of ZO-1 and Claudin-5 levels in the cortex of control and α5-PKO mice. *n* = 4. **d** TEM images showing normal tight junction structure in control and α5-PKO brains. Black arrowheads indicate tight junctions. TEM, transmission electron microscopy. Scale bar = 800 nm

BMECs express high levels of TJPs at the intercellular space forming tight junctions, which contribute to BBB integrity [3, 17, 78]. To determine if TJP expression is altered, we examined the levels of two TJPs, ZO-1 and claudin-5, in control and α5-PKO brains. Immunohistochemistry revealed similar distribution patterns of ZO-1 and claudin-5 in control and α5-PKO brains. Specifically, both proteins co-localized well with blood vessel marker CD31, in both cortex (Fig. 3a, b) and striatum (not shown). Similarly, western blotting was performed to quantify TJP expression in control and α5-PKO brains. No significant differences in ZO-1 and claudin-5 levels were observed between genotypes in either cortex (Fig. 3c) or striatum (not shown). Consistent with these biochemical findings, TEM study revealed no obvious defects in the structure of tight junctions (Fig. 3d, arrowheads). These results suggest that mural cell-derived laminin-α5 plays a dispensable role in the regulation of TJP expression and tight junction structure under homeostatic conditions.

### Pericyte coverage and astrocyte polarity are unaltered in α5-PKO mice under homeostatic conditions

**Fig. 4:**
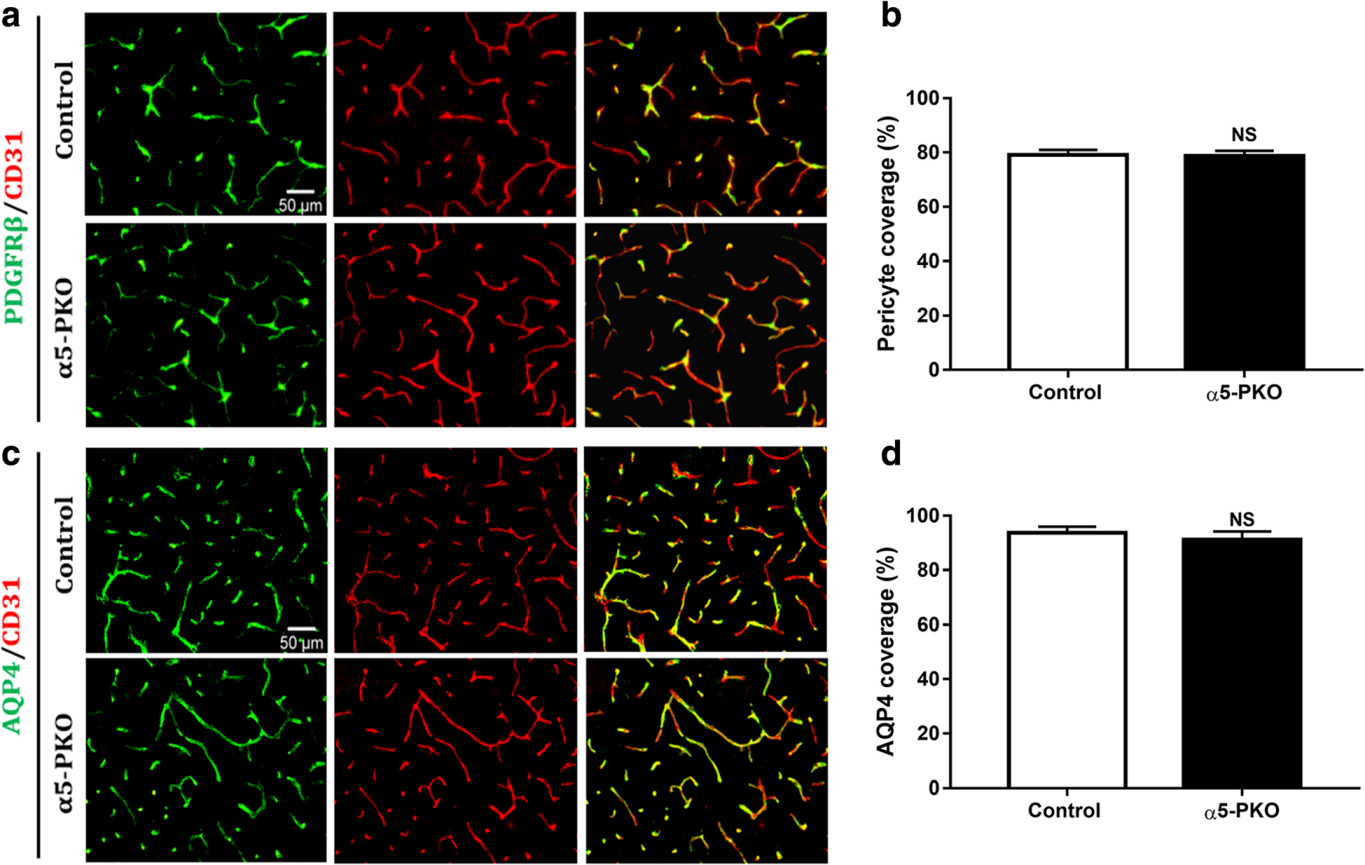
Pericyte coverage and AQP4 coverage are unaffected in α5-PKO mice under homeostatic conditions. **a** Representative images of PDGFRβ (green) and CD31 (red) staining in the cortex of control and α5-PKO mice. Scale bar = 50 μm. **b** Quantification showing comparable pericyte coverage in the cortex of control and α5-PKO mice. *n* = 4. **c** Representative images of AQP4 (green) and CD31 (red) staining in the cortex of control and α5-PKO mice. Scale bar = 50 μm. **d** Quantification showing comparable AQP4 coverage in the cortex of control and α5-PKO mice. *n* = 4. AQP4, aquaporin-4

Pericyte coverage on capillaries plays an important role in maintaining BBB integrity [4, 5, 8, 18]. To determine if pericyte coverage is altered in α5-PKO brains, we performed immunohistochemistry against PDGFRβ and CD31 (Fig. 4a). Quantification revealed comparable pericyte coverage in control and α5-PKO mice in both cortex (Fig. 4b) and striatum (not shown), suggesting that pericyte coverage is unaffected in α5-PKO mice under homeostatic conditions.

Astrocytes express AQP4 exclusively in their endfeet, contributing to BBB maintenance [5, 26, 78]. To determine if the polarized distribution of AQP4 is altered in α5-PKO brains, we performed immunohistochemistry against AQP4 and CD31 (Fig. 4c). Quantification revealed comparable AQP4 coverage in control and α5-PKO mice in both cortex (Fig. 4d) and striatum (not shown), suggesting that astrocyte polarity is unchanged in α5-PKO mice under homeostatic conditions.

### α5-PKO mice have smaller infarct volume and improved neurological function after ischemia-reperfusion injury

**Fig. 5:**
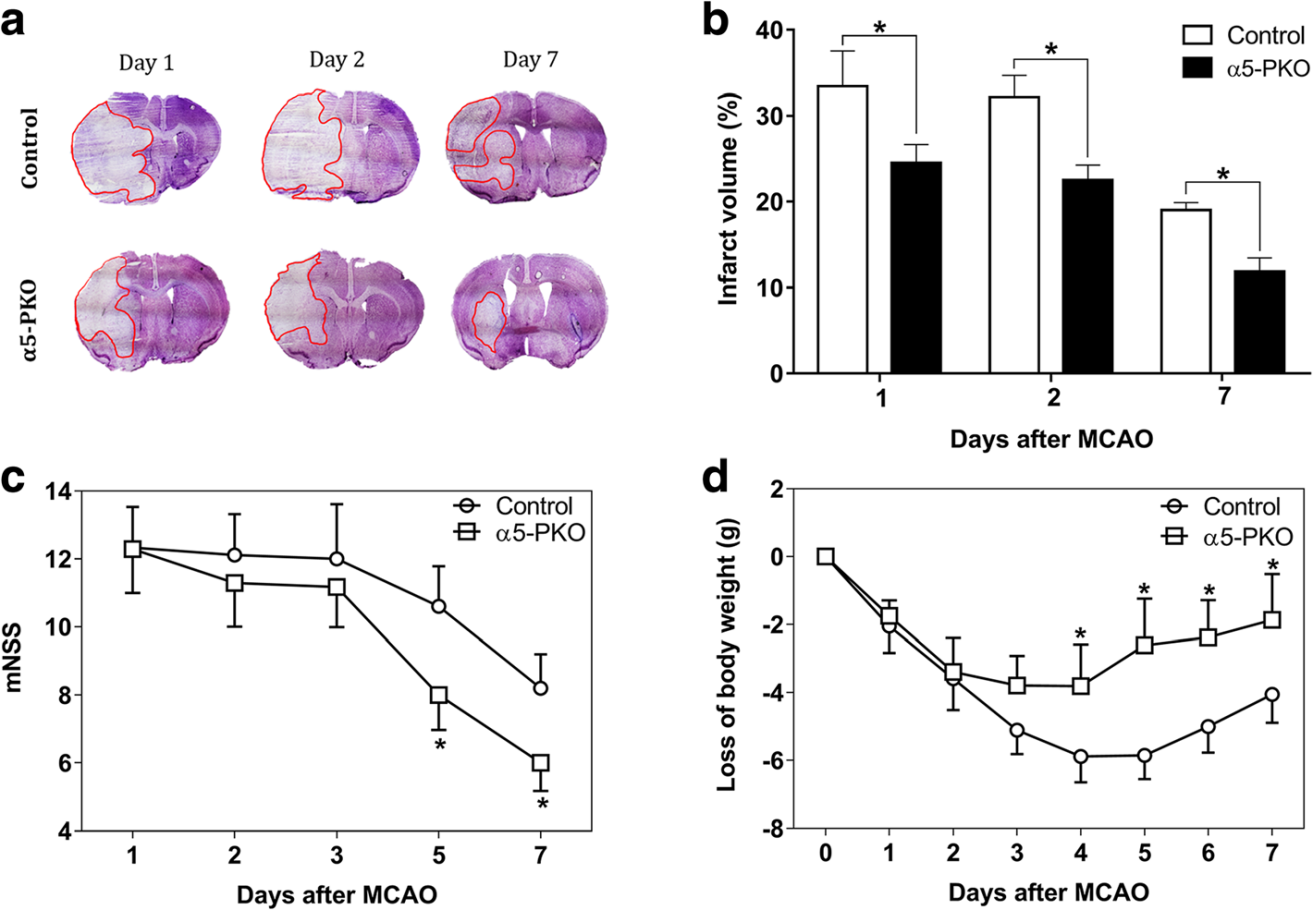
α5-PKO mice have smaller injury size, improved neurological function and alleviated body weight loss after ischemic stroke. **a** Representative images of cresyl violet staining in control and α5-PKO brains at days 1, 2 and 7 after injury. **b** Quantification showing reduced brain infarct volume in α5-PKO mice at days 1, 2 and 7 after injury. *n* = 8. **c** Quantification showing decreased neurological severity score in α5-PKO mice at days 5 and 7 after injury. mNSS, modified neurological severity score. *n* = 8. **d** Quantification showing attenuated body weight loss in α5-PKO mice at days 4–7 after injury. *n* = 8. *p < 0.05, compared to controls at the same time points

To investigate the function of mural cell-derived laminin-α5 in ischemia-reperfusion injury, we first examined brain infarct volume at various time points after MCAO. Control mice demonstrated large infarct volume at days 1 and 2 after injury, which was reduced dramatically by day 7 after injury (Fig. 5a). A similar trend was observed in α5-PKO mice (Fig. 5a). Quantification revealed significantly smaller infarct volume (Fig. 5b) in α5-PKO mice at all three time points compared to the controls, suggesting reduced ischemic injury. To visualize the spatial distribution of infarct areas in control and α5-PKO brains at day 1 after injury, 5 brain sections along the rostral-to-caudal axis (with equal distance) were used for analyses. Similarly, α5-PKO mice demonstrated diminished infarct volume compared to the controls (Additional file 1: Figure S2). Consistent with the reduced infarct volume, significantly lower neurological severity score was detected in α5-PKO mice at days 5 and 7 after injury (Fig. 5c), indicating improved neurological function. In addition, the α5-PKO mice also displayed substantially less body weight loss at days 4–7 after injury (Fig. 5d). Together, these results suggest better pathological and functional outcomes in α5-PKO mice after ischemia-reperfusion injury. To determine any gender differences, these parameters were also analyzed in a gender-specific manner. Compared to male mice, female mice showed smaller infarct volume, lower neurological score, and less body weight loss independent of genotype, although these changes did not reach statistical significance (Additional file 1: Figure S3).

### α5-PKO mice have reduced neuronal death after ischemia-reperfusion injury

To investigate if loss of mural cell-derived laminin-α5 affects neuronal death after ischemic injury, we performed FJC staining, which labels degenerating neurons [11, 72]. FJC^+^ cells were identified in both penumbra (Additional file 1: Figure S4a) and ischemic core (Additional file 1: Figure S4c) in control and α5-PKO brains. Consistent with previous finding that FJC^+^ cells peak at 24 h after ischemic injury [41], quantitative data showed a continuous decline of FJC^+^ cell number in both penumbra (Additional file 1: Figure S4b) and ischemic core (Additional file 1: Figure S4d) in control mice from days 1 to 7 after ischemia-reperfusion injury. Although a similar trend was found in α5-PKO mice, these mutants showed significantly fewer FJC^+^ cells in both regions (Additional file 1: Figure S4) at all three time points compared to the controls. These results suggest reduced neuronal death in α5-PKO mice after ischemic injury.

### α5-PKO mice have milder BBB disruption after ischemia-reperfusion injury

**Fig. 6:**
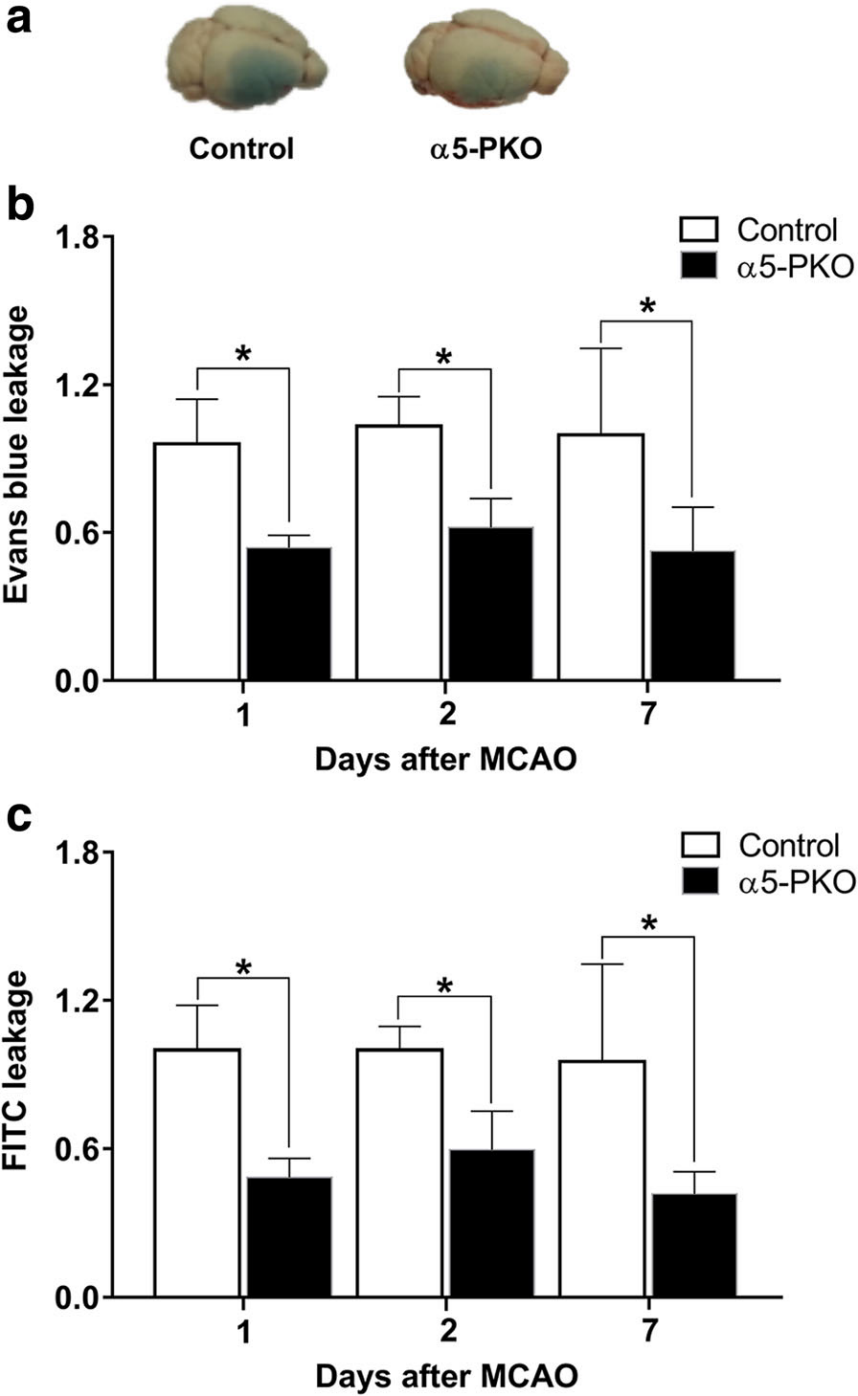
α5-PKO mice have milder BBB disruption after ischemic stroke. **a** Representative images showing Evans blue leakage in control and α5-PKO brains at day 1 after injury. **b** Quantification showing reduced Evans blue leakage in α5-PKO brains at day 1, 2 and 7 after injury. *n* = 5. **c** Quantification showing reduced FITC leakage in α5-PKO brains at day 1, 2 and 7 after injury. *n* = 5. *p < 0.05, compared to controls

BBB permeability was assessed by measuring EB and FITC-Dextran leakage at days 1, 2 and 7 after injury. Representative whole-brain images showing EB leakage in control and α5-PKO mice at day 1 after injury are shown in Fig. 6a. Compared to the controls, significantly reduced EB leakage was detected in α5-PKO mice at days 1, 2 and 7 after injury (Fig. 6b). Consistent with EB data, dramatically diminished FITC-Dextran leakage was found in α5-PKO mice at all three time points (Fig. 6c). These results suggest milder BBB damage in α5-PKO mice after ischemia-reperfusion injury.

### α5-PKO mice have decreased inflammatory cell infiltration after ischemia-reperfusion injury

**Fig. 7:**
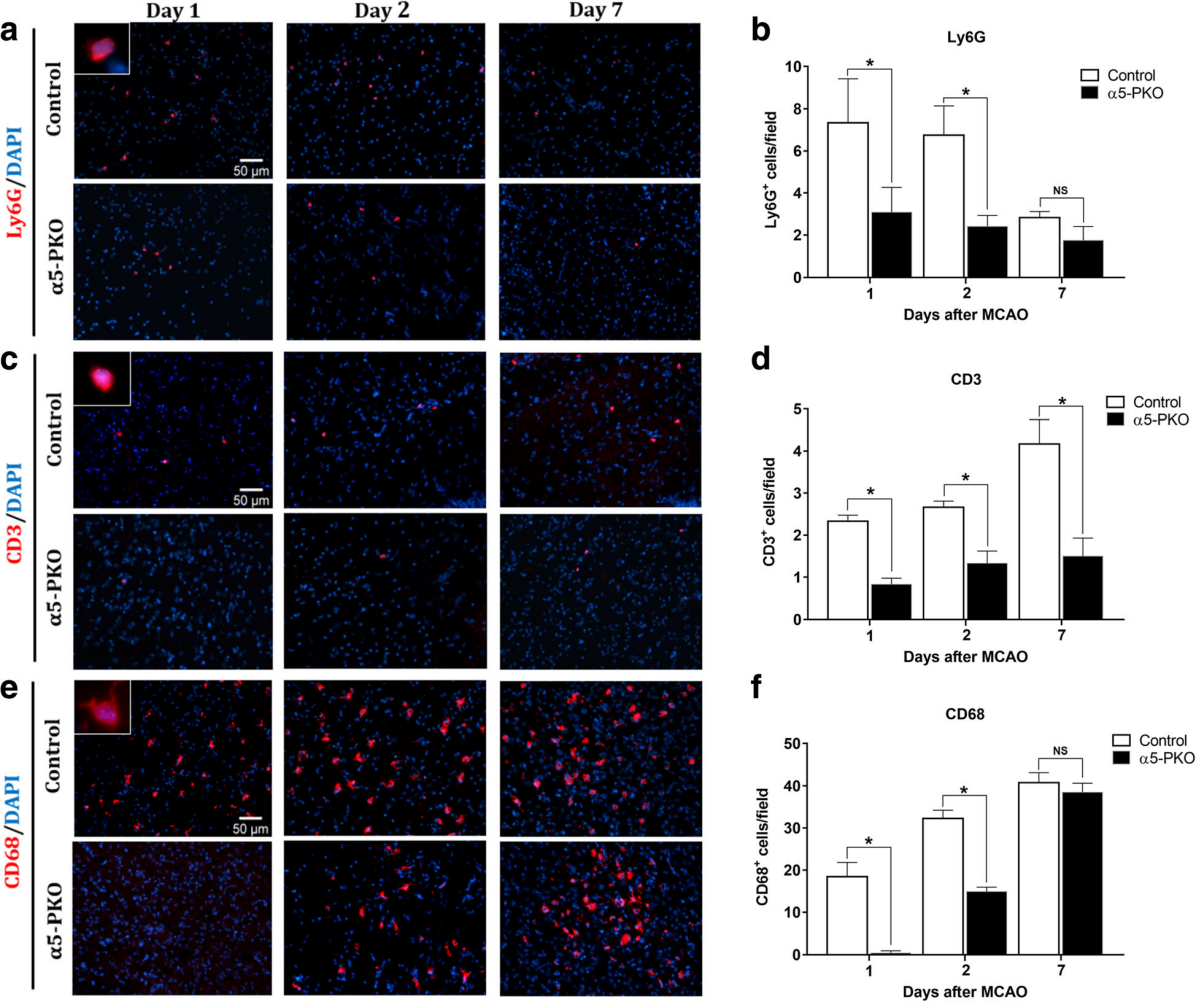
α5-PKO mice show decreased inflammatory cell infiltration after ischemic stroke. **a** Representative images of Ly6G (red) staining in the penumbra of control and α5-PKO brains at days 1, 2 and 7 after injury. Scale bar = 50 μm. **b** Quantification showing reduced extravasation of Ly6G^+^ neutrophils in the penumbra of α5-PKO brains at days 1 and 2 but not 7 after injury. *n* = 5. **c** Representative images of CD3 (red) staining in the penumbra of control and α5-PKO brains at days 1, 2 and 7 after injury. Scale bar = 50 μm. **d** Quantification showing reduced extravasation of CD3^+^ lymphocytes in the penumbra of α5-PKO brains at days 1, 2 and 7 after injury. *n* = 5. **e** Representative images of CD68 (red) staining in the penumbra of control and α5-PKO brains at days 1, 2 and 7 after injury. Scale bar = 50 μm. **f** Quantification showing reduced extravasation of CD68^+^ mononuclear cells in the penumbra of α5-PKO brains at days 1 and 2 but not 7 after injury. *n* = 5. * *p* < 0.05, compared to controls at the same time points

Accumulating evidence demonstrates that immune cells infiltrate into the brain and modulate disease progression after ischemic stroke [27, 31]. To investigate if inflammatory cell extravasation is affected in α5-PKO mice, we examined the infiltration of Ly6G^+^ neutrophils, CD3^+^ lymphocytes, and CD68^+^ mononuclear cells in both penumbra and ischemic core at days 1, 2, and 7 after injury. In control mice, the number of Ly6G^+^ neutrophils peaked at early time points (days 1 and 2) after injury and gradually declined over time in both penumbra (Fig. 7a, b) and ischemic core (Additional file 1: Figures S5a and b). Compared to the controls, α5-PKO mice showed significantly decreased Ly6G^+^ neutrophil number at days 1 and 2 but not 7 after injury in both penumbra (Fig. 7a, b) and ischemic core (Additional file 1: Figures S5a and b), suggesting diminished neutrophil infiltration in the mutants. Unlike Ly6G^+^ neutrophils, the number of CD3^+^ lymphocytes gradually increased over time after injury in control mice in both penumbra (Fig. 7c, d) and ischemic core (Additional file 1: Figure S5c and d). Compared to the controls, α5-PKO mice displayed substantially less CD3^+^ lymphocytes in both penumbra (Fig. 7c, d) and ischemic core (Additional file 1: Figure S5c and d) at all three time points, suggesting decreased lymphocyte infiltration in the mutants. Similar to CD3^+^ lymphocytes, the number of CD68^+^ mononuclear cells gradually elevated overtime after injury in control mice in both penumbra (Fig. 7e, f) and ischemic core (Additional file 1: Figure S5e and f). Compared to the controls, α5-PKO mice demonstrated dramatically reduced CD68^+^ mononuclear cell number at days 1 and 2 but not 7 after injury in both penumbra (Fig. 7e, f) and ischemic core (Additional file 1: Figure S5e and f), suggesting attenuated mononuclear cell infiltration in the mutants. Altogether, these data suggest reduced inflammatory cell extravasation in α5-PKO mice after ischemia-reperfusion injury.

### α5-PKO mice have less severe TJP loss after ischemia-reperfusion injury

**Fig. 8:**
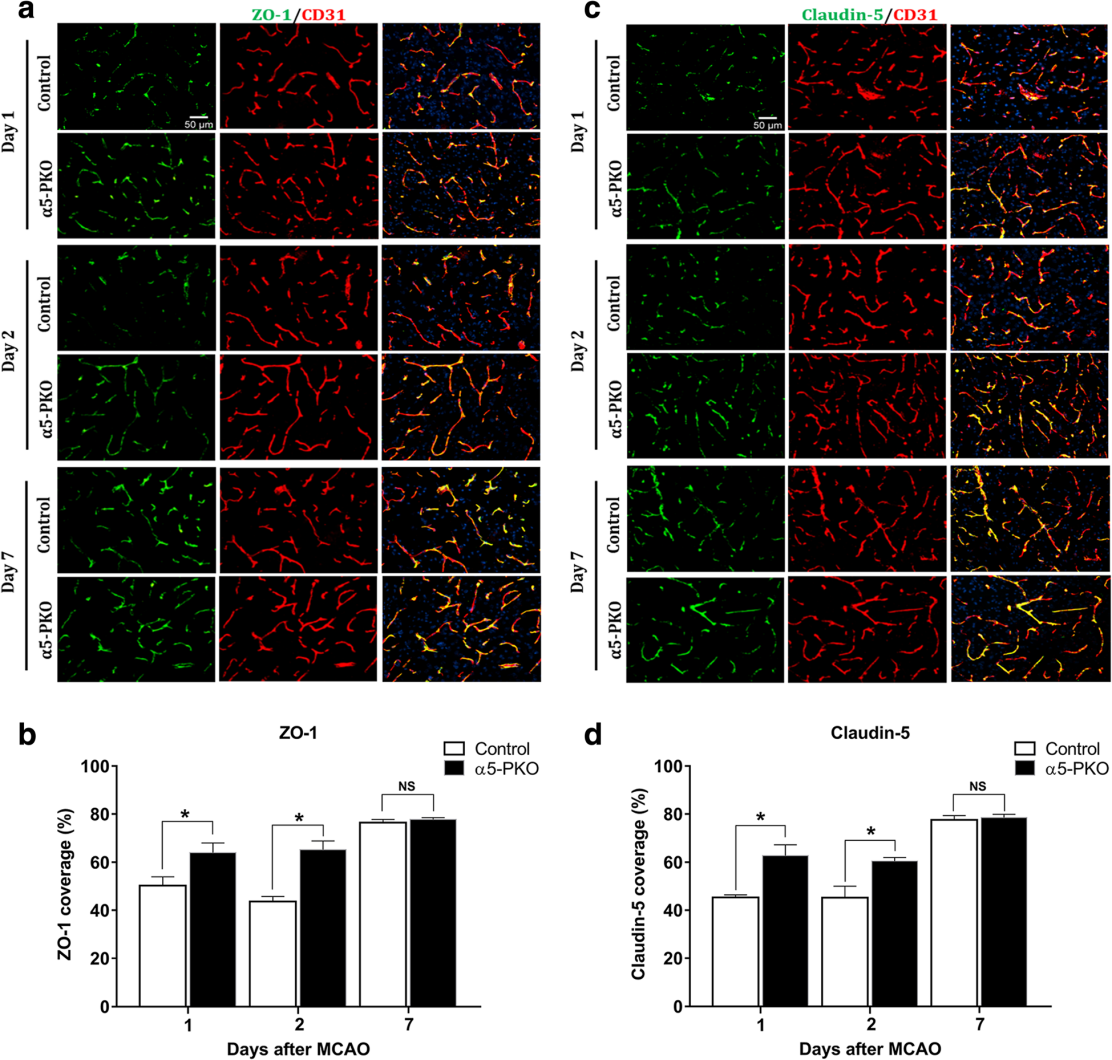
α5-PKO mice have less severe TJP loss after ischemic stroke. **a** Representative images of ZO-1 (green) and CD31 (red) staining in the penumbra of control and α5-PKO brains at days 1, 2 and 7 after injury. Scale bar = 50 μm. **b** Quantification showing higher ZO-1 coverage in the penumbra of α5-PKO brains at days 1 and 2 but not 7 after injury. *n* = 5. **c** Representative images of Claudin-5 (green) and CD31 (red) staining in the penumbra of control and α5-PKO brains at days 1, 2 and 7 after injury. Scale bar = 50 μm. **d** Quantification showing higher Claudin-5 coverage in the penumbra of α5-PKO brains at days 1 and 2 but not 7 after injury. *n* = 5. * *p* < 0.05, compared to controls at the same time points

To explore the molecular mechanisms responsible for the attenuated BBB disruption in α5-PKO mice, we first examined the dynamic changes of TJPs after ischemia-reperfusion injury. Immunohistochemistry showed substantial and mild loss of ZO-1 in CD31^+^ blood vessels in control mice early (at days 1 and 2) and late (at day 7) after ischemic injury, respectively (Fig. 8a). Although a similar trend was found in α5-PKO mice (Fig. 8a), ZO-1 levels were significantly higher in these mutants compared to the controls at days 1 and 2 but not 7 after ischemic injury (Fig. 8b). Like ZO-1, claudin-5 was dramatically reduced at days 1 and 2 after injury and slightly decreased at day 7 after injury in both control and α5-PKO mice (Fig. 8c). Compared to the controls, α5-PKO mice demonstrated significantly higher levels of claudin-5 at days 1 and 2 but not 7 after ischemic injury (Fig. 8d). These findings suggest that mural cell-derived laminin-α5 negatively regulates TJP expression early after ischemia-reperfusion injury.

### α5-PKO mice have less severe pericyte coverage reduction after ischemia-reperfusion injury

**Fig. 9:**
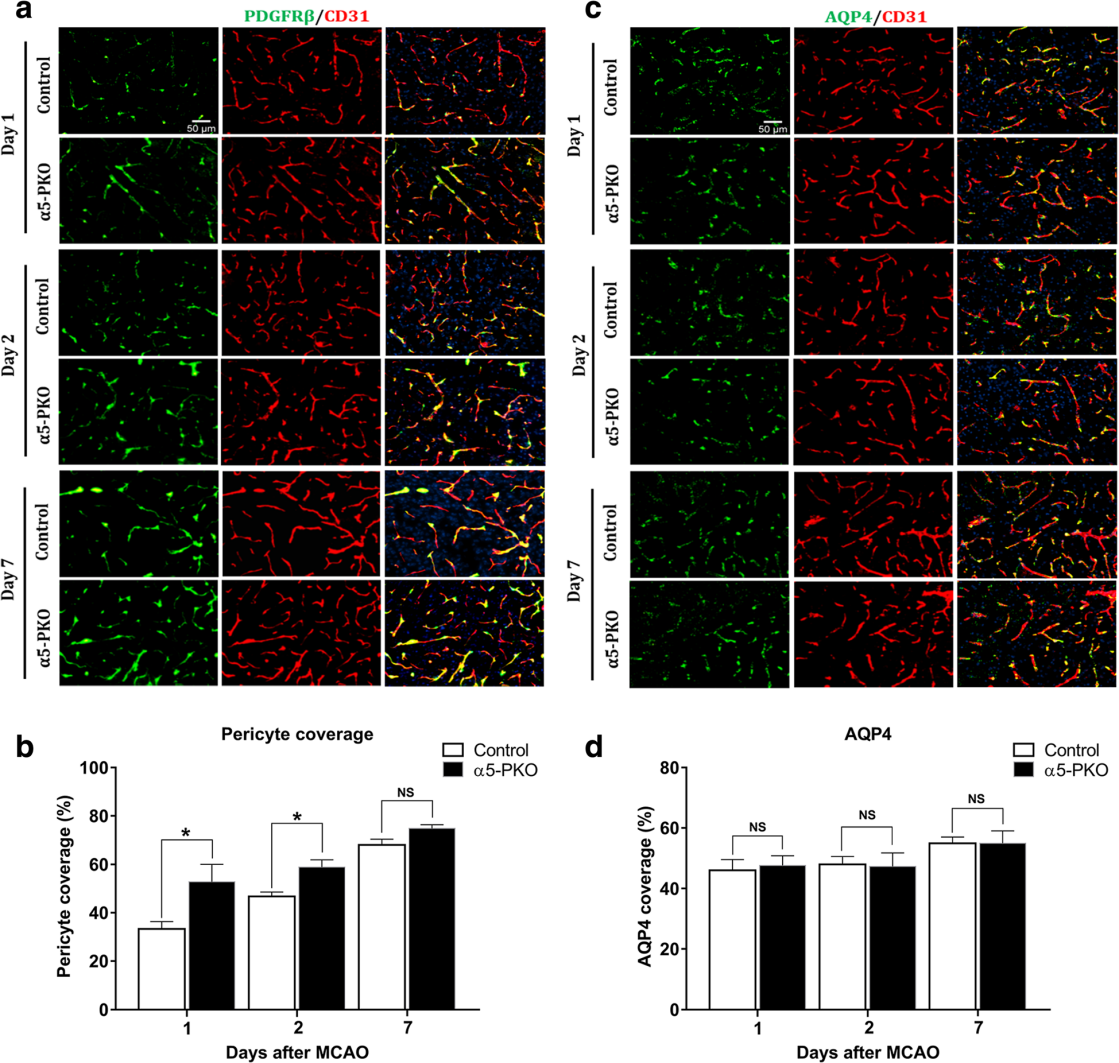
α5-PKO mice have less severe pericyte coverage reduction and unchanged AQP4 coverage after ischemic stroke. **a** Representative images of PDGFRβ (green) and CD31 (red) staining in the penumbra of control and α5-PKO brains at days 1, 2 and 7 after injury. Scale bar = 50 μm. **b** Quantification showing higher pericyte coverage in the penumbra of α5-PKO brains at days 1 and 2 but not 7 after injury. *n* = 5. **c** Representative images of AQP4 (green) and CD31 (red) staining in the penumbra of control and α5-PKO brains at days 1, 2 and 7 after injury. Scale bar = 50 μm. **d** Quantification showing similar AQP4 coverage in the penumbra of control and α5-PKO brains at days 1, 2 and 7 after injury. *n* = 5. * *p* < 0.05, compared to controls at the same time points. AQP4, aquaporin-4

Next, we examined the dynamic changes of pericyte coverage in control and α5-PKO mice after ischemia-reperfusion injury. Immunohistochemistry showed that both PDGFRβ intensity (Fig. 9a) and pericyte coverage (Fig. 9b) were substantially decreased in control mice at day 1 after ischemic injury compared to uninjured mice (see Fig. 4a, b). Pericyte coverage gradually recovered from day 1 to day 7 after injury in control mice (Fig. 9b). Although a similar trend was observed in α5-PKO mice (Fig. 9a), these mutants displayed significantly higher pericyte coverage at days 1 and 2 but not 7 after injury compared to the controls (Fig. 9b). These results suggest that mural cell-derived laminin-α5 negatively regulates pericyte coverage early after ischemia-reperfusion injury.

### Astrocyte polarity is unaffected in α5-PKO mice after ischemia-reperfusion injury

In addition, we also examined AQP4 expression with respect to CD31^+^ blood vessels in control and α5-PKO mice after ischemia-reperfusion injury. Neither its expression pattern nor expression level showed significant changes in α5-PKO mice after injury (Fig. 9c, d), suggesting that mural cell-derived laminin-α5 does not regulate astrocyte polarity under ischemia-reperfusion condition.

### α5-PKO mice have diminished brain edema after ischemia-reperfusion injury

**Fig. 10:**
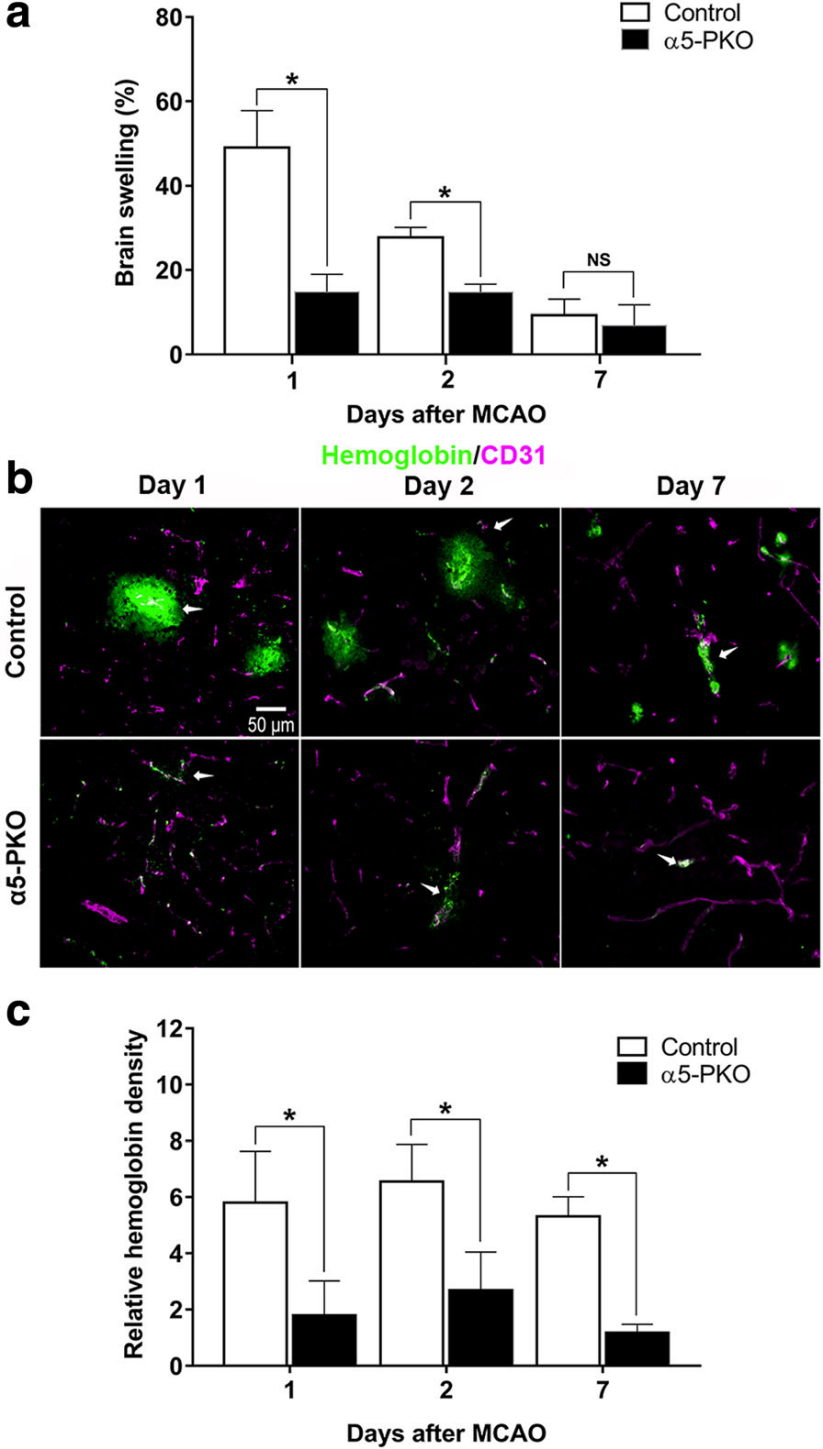
α5-PKO mice have diminished brain swelling and attenuated hemorrhagic transformation after ischemic stroke. **a** Quantification showing reduced brain swelling in α5-PKO mice at days 1 and 2 but not 7 after injury. *n* = 5. **b** Representative images of hemoglobin (green) and CD31 (magenta) staining in control and α5-PKO mice at days 1, 2 and 7 after injury. Scale bar = 50 μm. **c** Quantification showing reduced hemoglobin density in α5-PKO mice at days 1, 2 and 7 after injury. *n* = 5. * *p* < 0.05, compared to controls at the same time points

Both brain water content and brain swelling were used to assess brain edema after injury. Compared to the contralateral side, the ipsilateral side showed significantly higher water content in both control and α5-PKO mice at days 1 and 2 after injury (Additional file 1: Figure S6), indicating successful induction of ischemic injury. Comparison between genotypes revealed a dramatically reduced brain water content in the ipsilateral side in α5-PKO mice at day 1 after injury (Additional file 1: Figure S6). Although not statistically significant, a similar trend was observed at day 2 after injury (Additional file 1: Figure S6). By day 7 after injury, no significant differences in brain water content were found between hemispheres or between genotypes (Additional file 1: Figure S6), indicating successful recovery in both control and α5-PKO mice. When brain swelling was used to assess brain edema, statistical significance was detected between control and α5-PKO mice at days 1 and 2 but not 7 after injury (Fig. 10a). This difference is due to the fact that brain swelling is much more sensitive to smaller changes in brain water level than brain water content [33]. These results strongly suggest that brain edema is less severe in α5-PKO mice after ischemia-reperfusion injury.

### α5-PKO mice have alleviated hemorrhagic transformation after ischemia-reperfusion injury

Hemorrhagic transformation, a spectrum of an ischemia-related brain hemorrhage, is a frequent spontaneous complication of ischemic stroke [66]. To investigate if hemorrhagic transformation is altered in α5-PKO mice, hemorrhage in the ischemic core was examined at days 1, 2, and 7 after ischemic injury using hemoglobin staining. Strong hemoglobin signal was found in control mice, whereas weak staining was detected in α5-PKO mice at all time points (Fig. 10b). Quantification revealed significantly reduced hemoglobin intensity in α5-PKO mice at all three time points compared to the controls (Fig. 10c). These results suggest alleviated hemorrhagic transformation in α5-PKO mice after ischemia-reperfusion injury.

## Discussion

Mural cells include pericytes and vSMCs, which cover capillaries and arteries/arterioles, respectively [4]. Although it is known that mural cells are able to synthesize laminins, the exact laminin isoforms they make remain largely unknown. We have shown in a previous study that brain pericytes express γ1-containing laminins under homeostatic conditions [26]. Although laminin-α2 was reported in brain pericytes at mRNA level [5], our unpublished study showed that pericytes primarily make laminin-α4 and -α5 at protein level. Like pericytes, vSMCs also predominantly express laminin-α4 [30, 52, 69] and -α5 [46, 64, 65]. In addition, laminin-α2 has also been found in vSMCs from large vessels [45], such as the aorta and carotid arteries. There is also evidence showing that laminin-β1 is expressed in vSMCs in developing vessels, whereas laminin-β2 is found in mature vasculature [28], suggesting a switch from β1- to β2-containing laminins during vessel maturation. Collectively, these results suggest that mural cells mainly express laminin-411, − 511, and possibly − 211 during development; and laminin-421, − 521, and possibly − 221 in adulthood under homeostatic conditions.

In this study, we failed to detect any changes in BBB permeability and CBF between control and α5-PKO mice under physiological conditions, suggesting that mural cell-derived α5-containing laminins are dispensable for BBB maintenance and CBF regulation under homeostatic conditions. Unlike these α5-PKO mice, mutants with laminin-γ1 deficiency (all γ1-containing laminins) in mural cells showed BBB breakdown and hydrocephalus in C57Bl6-FVB mixed background [26], suggesting an important role of mural cell-derived γ1-containing laminins in BBB maintenance and hydrocephalus pathogenesis, although we cannot exclude the possibility that BBB disruption is secondary to hydrocephalus. Together, these findings suggest the existence of compensation between mural cell-derived α5-containing laminins and α4/α2-containing laminins. In addition, it is also possible that the lack of phenotype in α5-PKO mice under homeostatic conditions is due to compensation by laminin isoforms from endothelial cells and/or astrocytes, which are in close proximity of mural cells [67]. For example, mural cell-derived α5-containing laminins and endothelial cell-derived laminin-511 may be able to compensate for each other’s loss. In support of this possibility, mice with laminin-α5 deficiency in endothelial cells are grossly normal and fail to show any defects under homeostatic conditions [25, 63].

After ischemia-reperfusion injury, α5-PKO mice demonstrated alleviated BBB disruption at days 1, 2, and 7 after injury. Consistent with the reduced BBB leakage, TJP (ZO-1 and claudin-5) levels were less severely reduced in the mutants at days 1 and 2 after injury. By day 7 after injury, however, BBB leakage but not TJP expression showed a significant difference between genotypes. This finding suggests that TJPs are not responsible for the difference in BBB integrity between genotypes at this time point, highlighting a possible role of transcytosis in BBB integrity maintenance. Echoed with this observation, tight junction-independent BBB disruption and the important role of transcytosis in BBB regulation have been reported in recent studies [2, 10, 17, 36, 71].

In addition, α5-PKO mice also displayed diminished inflammatory cell (neutrophil, lymphocyte, and mononuclear cell) infiltration, suggesting a “pro-infiltration” role of mural cell-derived laminin-α5 after ischemic injury. This is in contrast with a previously reported “anti-infiltration” role of endothelial laminin-α5. It has been demonstrated that loss of laminin-α5 in endothelial cells increased immune cell extravasation in cremaster muscle after inflammation [63] and in the brain after ICH [25]. In addition, in the EAE model, reduced infiltration of T lymphocytes in the brain was found in laminin-α4 null mice, which demonstrated compensatory & ubiquitous expression of laminin-α5 in the vasculature [73]. One explanation for this discrepancy is that mural cells and endothelial cells make different α5-containing laminins, which exert distinct functions to regulate immune cell extravasation. It should be noted, however, that we cannot exclude the possibility that mural cells make “new” laminin isoforms after ischemic injury, which are responsible for the observed “pro-infiltration” effect. Another possibility is that different injury/animal models and time points are responsible for this discrepancy. The “anti-infiltration” role of endothelial laminin-α5 is mainly supported by studies using a muscle inflammation model [63], an ICH model [25], and an EAE model [73], whereas the “pro-infiltration” role of mural cell-derived laminin-α5 is obtained from ischemia-reperfusion study. Unlike ischemia-reperfusion injury, the muscle inflammation model does not damage the BBB or the CNS. Additionally, loss of endothelial laminin-α5-induced increase of immune cell infiltration only occurs at a specific time point (1.5 h after TNFα injection) in this muscle inflammation model [63]. Although BBB disruption is replicated in the ICH model, brain pathology in ICH is completely different from that in ischemia-reperfusion injury. For example, blood vessel wall and BM are disrupted in the collagenase-induced ICH model, which causes immediate leakage of inflammatory cells into the brain, whereas such vascular damage is absent in the ischemia-reperfusion model. Therefore, it is unclear whether the increased accumulation of inflammatory cells in mutant brains is due to a direct “anti-infiltration” effect of endothelium-derived laminin-α5. In the EAE model, laminin-α4 global knockouts that showed compensatory up-regulation of laminin-α5 rather than endothelium-specific laminin-α5 knockouts were used [73]. Since mural cells also synthesize α4-containing laminins [30, 52, 69], both endothelium- and mural cell-derived laminin-α4 is ablated in these laminin-α4 knockouts. It is thus unclear whether the enhanced laminin-α5 is from endothelial cells or mural cells, which makes data interpretation difficult. We are currently investigating the role of endothelium-derived laminin-α5 in ischemic stroke using endothelium-specific laminin-α5 conditional knockout mice. Results from this study will contribute to our understanding of the biological function of endothelial laminin-α5.

α5-PKO mice exhibited milder vascular damage, such as less severe BBB disruption and decreased inflammatory cell infiltration, and attenuated neurological injury, including reduced ischemic volume, diminished neuronal death, and improved neurological function. Given that inflammatory cells actively contribute to secondary brain injury after stroke [1], we speculate that the attenuated neurological injury is due to milder vascular damage. In support of this possibility, extravasated neutrophils have been demonstrated to contribute to neuronal injury and brain edema in ischemic injury [12, 34, 37, 55, 60]. Similarly, lymphocytes are found to be responsible for delayed post-ischemic injury [39, 40]. In addition, monocytes have been shown to play a detrimental role in the acute phase (up to 3 days) after ischemic injury, although a beneficial role is reported in the chronic phase (after day 3) [21, 22]. Consistent with these reports, reduced numbers of neutrophils, lymphocytes, and mononuclear cells were observed in α5-PKO mice after ischemic injury, especially at early time points. It should be noted, however, that we are unable to exclude the possibility that attenuated neurological injury leads to milder vascular damage.

α5-PKO mice demonstrate a better outcome after ischemia-reperfusion injury, suggest a detrimental role of mural cell-derived laminin-α5 in ischemic injury. Similar to our α5-PKO mutants, mice with endothelium-specific deletion of integrin-α5 demonstrated substantially reduced infarct size, increased BBB integrity and improved neurological function after stroke [54], highlighting an adverse effect of endothelial integrin-α5 in ischemic stroke. Together, these findings suggest that mural cell-derived α5-containing laminins and endothelial integrin-α5 may use a converging signaling pathway to modulate the development/progression of ischemic stroke, although integrin-α5 is not a classical laminin receptor [6, 74]. Identifying the receptors and downstream signaling pathways may provide innovative molecular targets with therapeutic potential in ischemic stroke**.**

Due to the multiphasic nature of ischemic stroke, this study has a few limitations. First, only the transient ischemic model was used in this study. The transient ischemic model involves both ischemia and reperfusion. However, most strokes found in human patients only involve ischemia without reperfusion [19, 23, 42, 61, 70]. Thus, it is important to test the biological function of mural cell-derived laminin-α5 in the permanent ischemic model. Second, only one ischemic duration (45 min) was used in this study. It is known that longer occlusion causes more severe injury [44, 49]. Currently, various ischemic durations ranging from 30 to 120 min have been used in rodent MCAO studies [13, 14, 35, 49]. We chose 45-min ischemia for two reasons: (1) compared to other durations, 45-min ischemia consistently induced significant ischemic injury with less mortality in our hands, and (2) significant differences in stroke outcomes between control and α5-PKO mice were observed with 45-min ischemia. Other ischemic durations should be tested in future studies. Third, only young mice were used in this study. Aging is a risk factor for ischemic stroke and actively influences stroke outcomes [19, 23, 42]. Therefore, it is important to examine the biological function of mural cell-derived laminin-α5 in ischemic stroke using aged mice in the future. Fourth, unlike previous studies reporting improved outcomes in young female mice [19, 23, 42], we failed to observe gender differences in infarct volume, neurological severity score, and body weight loss. It should be noted that, although not statistically significant, a trend toward attenuated injury was observed in female mice independent of genotype. This discrepancy may be explained by the relatively small animal number used in each group and/or other factors, such as the severity of injury and sensitivity of assays. Future research is needed to address these limitations.

## Conclusions

Collectively, our results suggest that mural cell-derived laminin-α5 is dispensable for BBB maintenance and CBF regulation under homeostatic condition. In ischemic stroke, however, loss of mural cell-derived laminin-α5 attenuates vascular damage and improves stroke outcomes, indicating a detrimental role of mural cell-derived laminin-α5 in ischemic stroke. These findings identify mural cell-derived laminin-α5 as a molecular target with therapeutic potential in ischemic stroke.

## Additional file

Additional file 1: **Table S1.** Modified Neurologic Severity Scores (mNSS) system. **Figure S1.** Angioarchitecture is unaltered in α5-PKO mice under homeostatic conditions. **Figure S2.** Spatial distribution of infarct area in control and α5-PKO mice at day 1 after ischemic injury. **Figure S3.** Comparison of gender-specific effects in control and α5-PKO mice after ischemic stroke. **Figure S4.** α5-PKO mice show reduced neuronal death in both penumbra and ischemic core after ischemic stroke. **Figure S5.** α5-PKO mice have reduced inflammatory cell infiltration after ischemic stroke. **Figure S6.** α5-PKO mice have reduced brain water content after ischemic stroke. (DOCX 2830 kb)

## Abbreviations

BBB: Blood-brain barrier
BM: Basement membrane
BMECs: Brain microvascular endothelial cells
CBF: Cerebral blood flow
CCA: Common carotid artery
CNS: Central nervous system
EAE: Experimental autoimmune encephalomyelitis
ECA: External carotid artery
ICA: Internal carotid artery
ICH: Intracerebral hemorrhage
MCAO: Middle cerebral artery occlusion
SMA: Smooth muscle actin-α
TEM: Transmission electron microscopy
TJP: Tight junction protein
vSMCs: Vascular smooth muscle cells
