# Supplementary material for: Mural cell-derived laminin-α5 plays a detrimental role in ischemic stroke
Source: Acta Neuropathol Commun. 2019 Feb 18;7:23. doi: 10.1186/s40478-019-0676-8 (PMC6378751; doi:10.1186/s40478-019-0676-8)
Supplement: Supplementary file 1 — Table S1. Modified Neurologic Severity Scores (mNSS) system. Figure S1. Angioarchitecture is unaltered in α5-PKO mice under homeostatic conditions. Figure S2. Spatial distribution of infarct area in control and α5-PKO mice at day 1 after ischemic injury. Figure S3. Comparison of gender-specific effects in control and α5-PKO mice after ischemic stroke. Figure S4. α5-PKO mice show reduced neuronal death in both penumbra and ischemic core after ischemic stroke. Figure S5. α5-PKO mice have reduced inflammatory cell infiltration after ischemic stroke. Figure S6. α5-PKO mice have reduced brain water content after ischemic stroke. (DOCX 2830 kb) [file 40478_2019_676_MOESM1_ESM.docx]

**Mural cell-derived laminin-α5 plays a detrimental role in ischemic stroke**

Abhijit Nirwane^1^, Jessica Johnson^1^, Benjamin Nguyen^1^, Jeffrey H. Miner^2^, Yao Yao^1,*^

^1^Department of Pharmaceutical and Biomedical Sciences, University of Georgia, 240 W Green Street, Athens, GA, USA

^2^Division of Nephrology, Department of Medicine, Washington University School of Medicine, St. Louis, MO, USA

*Corresponding Author:

Yao Yao, Ph.D., Department of Pharmaceutical and Biomedical Sciences, University of Georgia, 240 W Green Street, Athens, GA, 30602, USA. Tel: 706-542-1430; Fax: 706-542-5358; Email: [yyao@uga.edu](mailto:yyao@uga.edu)

Journal Name: Acta Neuropathologica Communications

**Table S1** Modified Neurologic Severity Scores (mNSS) system

| **Motor tests** | **Points** |
| --- | --- |
| *Raising the mouse by the tail*   - 1 Flexion of forelimb - 1 Flexion of hindlimb - 1 Head moved more than 10 degrees to the vertical axis within 30 seconds | 3 |
| *Walking on the floor (normal=0; maximum=3)*   - 0 Normal walk - 1 Inability to walk straight - 2 Circling toward the paretic side - 3 falling down to the paretic side | 3 |
| *Beam balance tests (normal=0; maximum=6)*   - 0 Balances with steady posture - 1 Grasps side of beam - 2 Hugs the beam and one limb falls down from the beam - 3 Hugs the beam and two limbs fall down from the beam, or spins on beam (>30 seconds) - 4 Attempts to balance on the beam but falls off (>20 seconds) - 5 Attempts to balance on the beam but falls off (>10 seconds) - 6 Falls off: No attempt to balance or hang onto the beam (<10 seconds) | 6 |
| *Reflexes absence*   - 1 Pinna reflex (a head shake when touching the auditory meatus) - 1 Corneal reflex (an eye blink when lightly touching the cornea with cotton) | 2 |
| Maximum points | 14 |

**Supplementary Figures and Figure Legends**

**
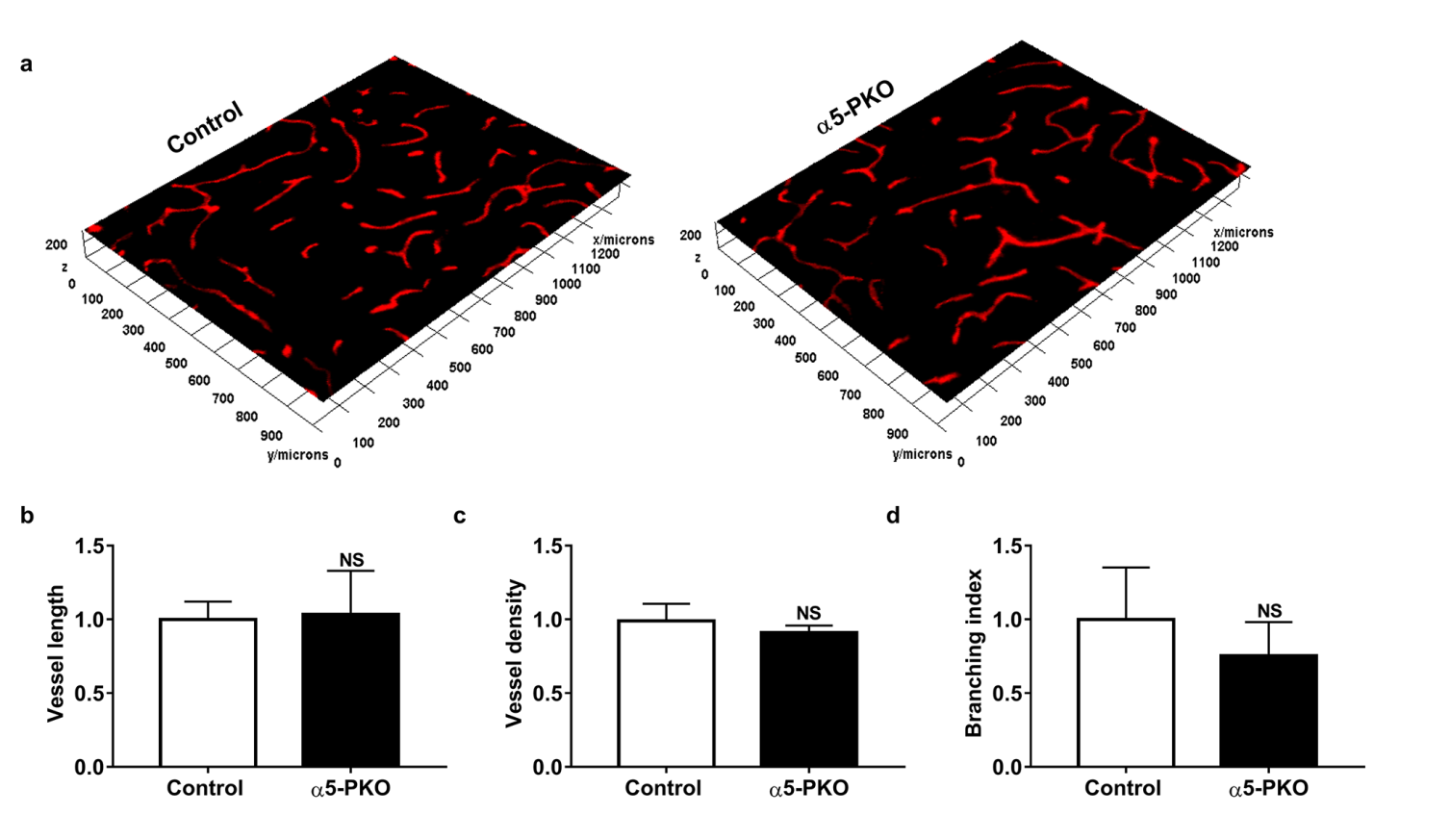
**

**Figure S1** Angioarchitecture is unaltered in α5-PKO mice under homeostatic conditions. **a** Representative images of CD31 (red) staining in control and α5-PKO brains. **b-d** Quantifications showing comparable vessel length (**b**), vessel density (**c**), and branching index (**d**) in control and α5-PKO brains. *n*=4.


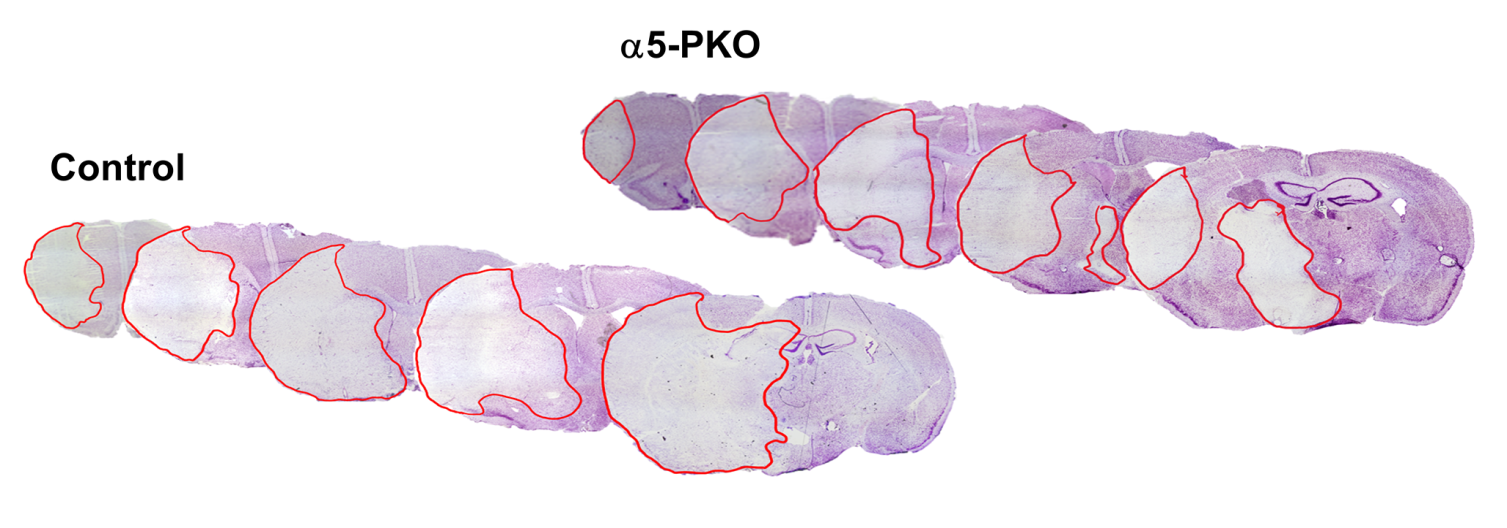


**Figure S2** Spatial distribution of infarct area in control and α5-PKO mice at day 1 after ischemic injury. Representative images of cresyl violet staining from 5 series sections along the rostral-to-caudal axis (with equal distance) in control and α5-PKO brains at day 1 after ischemic injury.

**
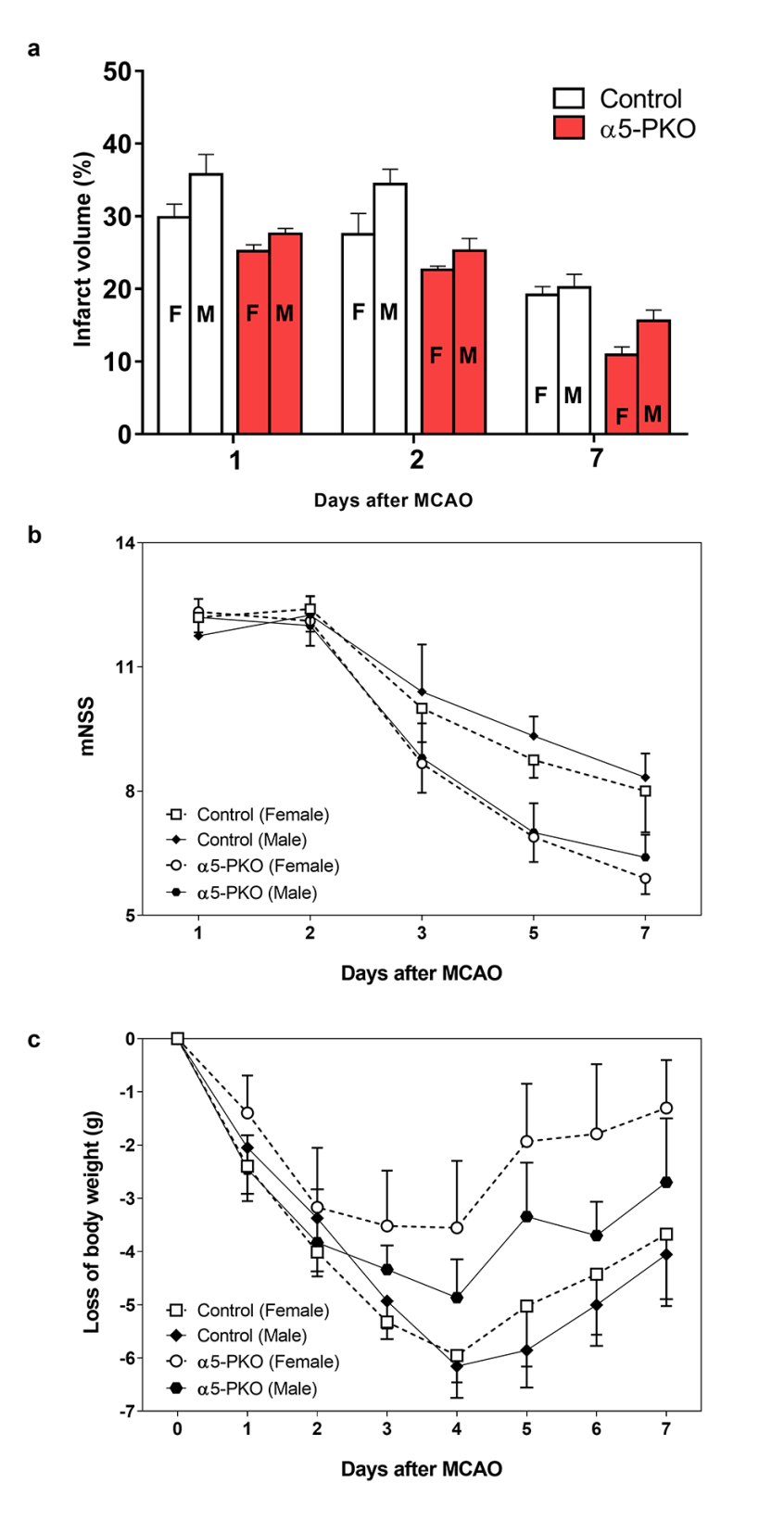
**

**Figure S3** Comparison of gender-specific effects in control and α5-PKO mice after ischemic stroke. **a-c** Compared to male mice, female mice displayed attenuated but not statistically significant changes in infarct volume (**a**), neurological severity score (**b**), and body weight loss (**c**) after ischemic stroke. *n*=4.

**
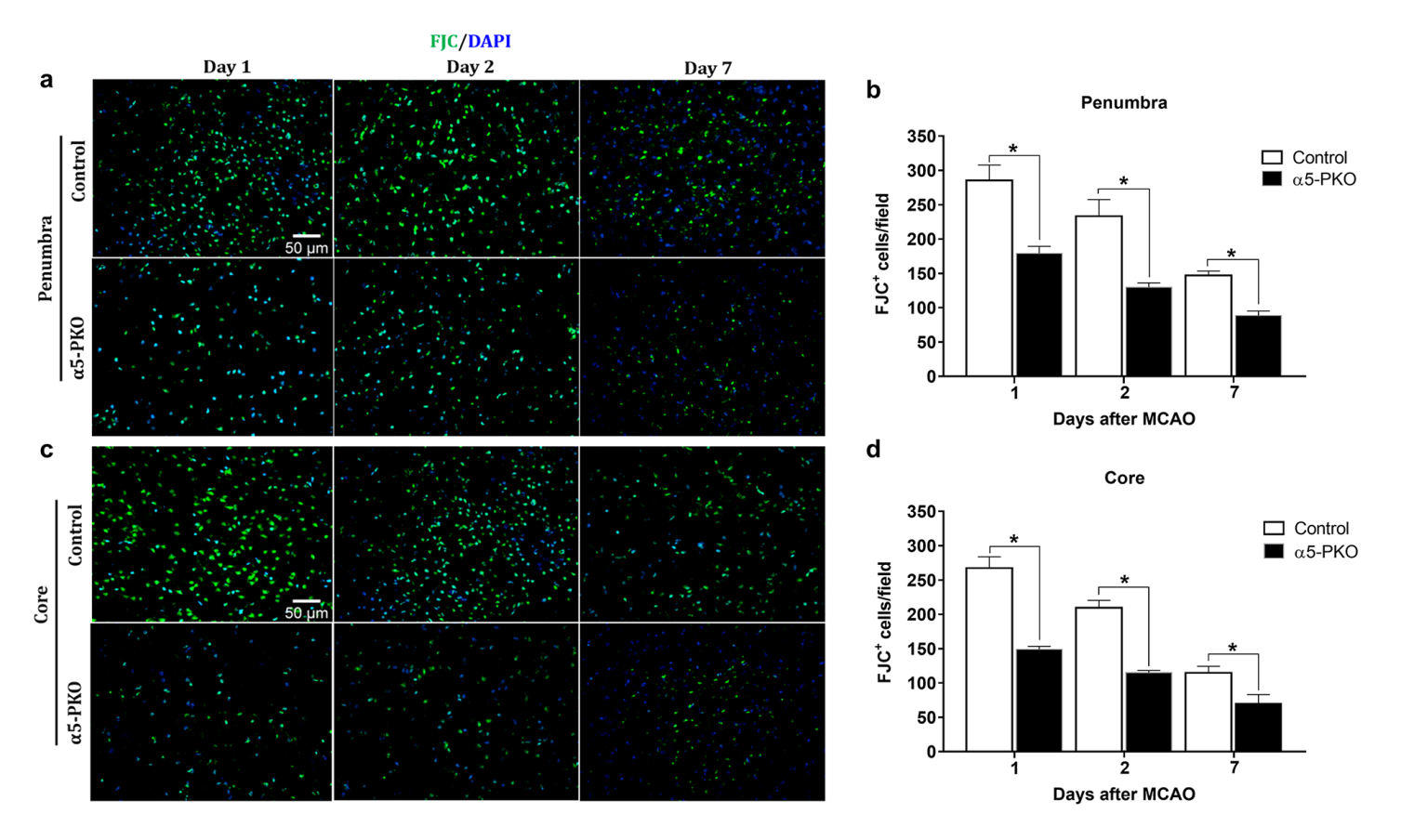
**

**Figure S4** α5-PKO mice show reduced neuronal death in both penumbra and ischemic core after ischemic stroke. **a and c** Representative images of FJC (green) staining in penumbra (**a**) and ischemic core (**c**) of control and α5-PKO brains at days 1, 2 and 7 after injury. Scale bar = 50μm. **b and d** Quantification showing diminished FJC^+^ cells in the penumbra (**b**) and ischemic core (**d**) of α5-PKO brains at days 1, 2 and 7 after injury. *n*=4. **p* < 0.05, compared to the controls at the same time points.


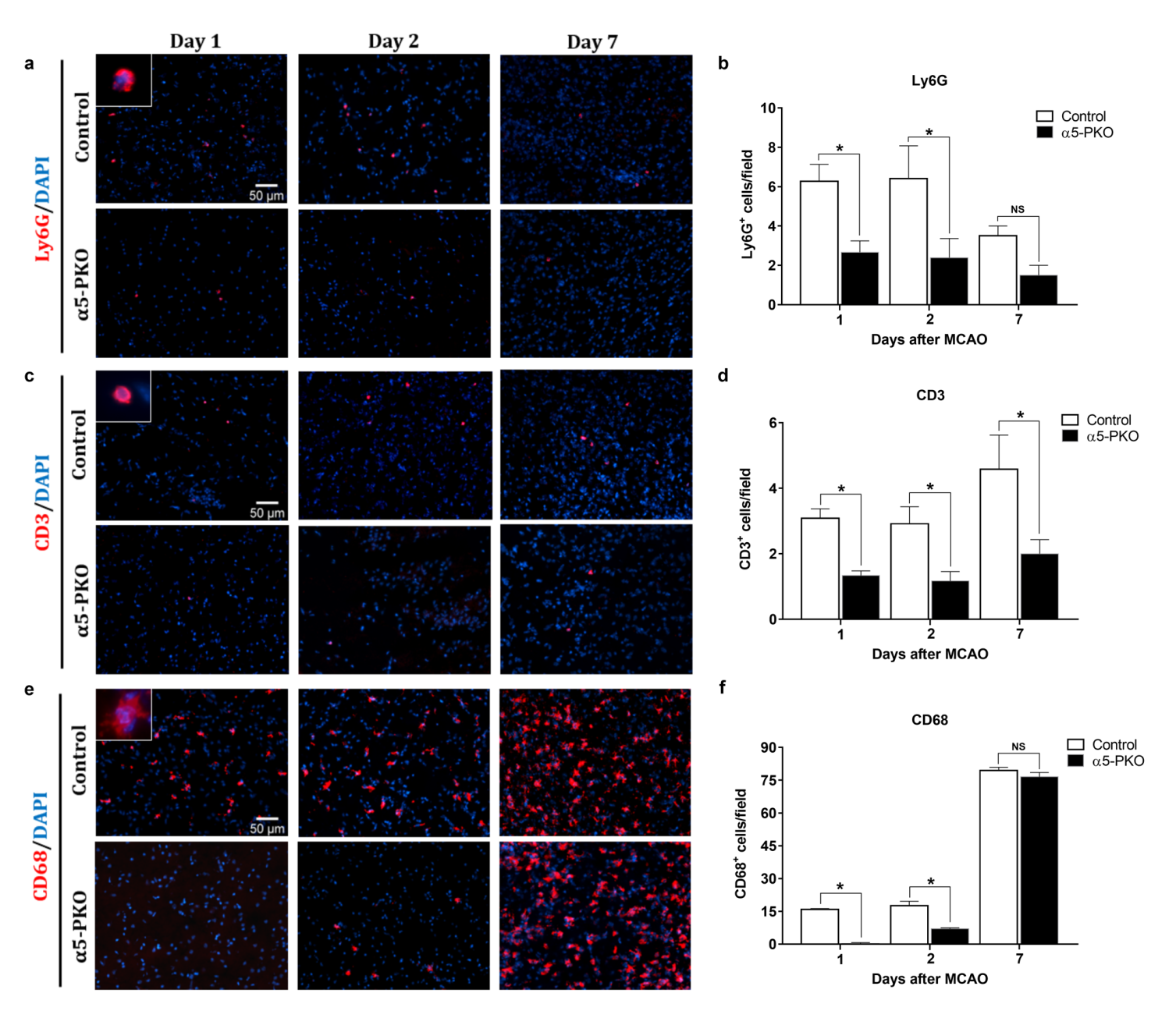


**Figure** **S5** α5-PKO mice have reduced inflammatory cell infiltration after ischemic stroke. **a** Representative images of Ly6G (red) staining in ischemic core of control and α5-PKO brains at days 1, 2 and 7 after injury. Scale bar = 50μm. **b** Quantification showing reduced extravasation of Ly6G^+^ neutrophils in ischemic core of α5-PKO brains at days 1 and 2 but not 7 after injury. *n*=5. **c** Representative images of CD3 (red) staining in ischemic core of control and α5-PKO brains at days 1, 2 and 7 after injury. Scale bar = 50μm. **d** Quantification showing reduced extravasation of CD3^+^ lymphocytes in ischemic core of α5-PKO brains at days 1, 2 and 7 after injury. *n*=5. **e** Representative images of CD68 (red) staining in ischemic core of control and α5-PKO brains at days 1, 2 and 7 after injury. Scale bar = 50μm. **f** Quantification showing reduced extravasation of CD68^+^ mononuclear cells in ischemic core of α5-PKO brains at days 1 and 2 but not 7 after injury. *n*=5. * *p* < 0.05, compared to controls at the same time points.


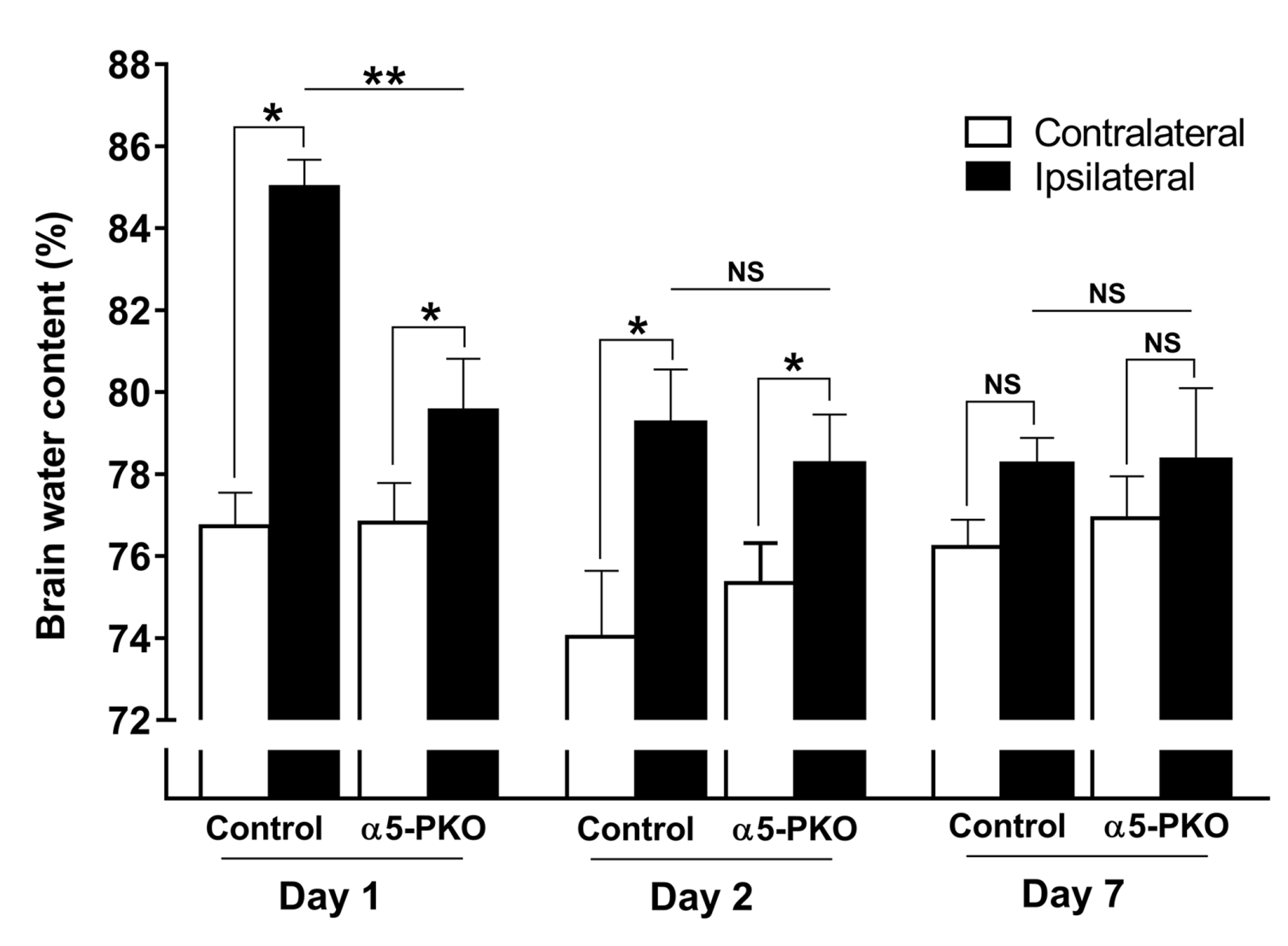


**Figure S6** α5-PKO mice have reduced brain water content after ischemic stroke. Quantification showing decreased brain water content in the ipsilateral side of α5-PKO brains at day 1, but not 2 or 7 after injury. *n*=5. ***p* < 0.05, compared to controls; **p* < 0.05, compared to contralateral hemispheres within the same genotypes.
